# Supplementary material for: Normalization by orientation-tuned surround in human V1-V3
Source: PLoS Comput Biol. 2023 Dec 27;19(12):e1011704. doi: 10.1371/journal.pcbi.1011704 (PMC10793941; doi:10.1371/journal.pcbi.1011704)

Data Set and Stimulus Properties

Table A: Data set properties for all experiments.

	url	Number of fMRI runs	V1 voxel count	V2 voxel count	V3 voxel count	voxel selection
**DS1**	https://osf.io/nm58r/	12	606	545	572	cross-validated $R^2 > 3\%$
**DS2**	https://osf.io/nxf65/	12	454	437	377	cross-validated $R^2 > 3\%$
**DS3**	https://osf.io/953yq/	24	202	283	215	cross-validated $R^2 >$ 0%,$mean(\beta) > 0$
**DS4**	https://osf.io/953yq/	12	189	163	227	cross-validated $R^2 >$ 0%,$mean(\beta) > 0$

DS1 = data set 1; DS2 = data set 2; DS1 = data set 1; DS2 = data set 2.

Table B: Stimulus set properties for all experiments

	url	Size (deg)	Size (pixels, shown to subjects)	Size (pixels, for analysis, excluding padding)	Spatial frequency (cpd)	Number of stimulus classes
**DS1**	https://osf.io/4u2er	12.5 × 12.5	400 × 400	150 × 150	3	50
**DS2**	https://osf.io/pnwgs	18.75 × 18.75	600 × 600	225 × 225	3	48
**DS3**	https://osf.io/dyx2q	12.5 × 12.5	800 × 800	150 × 150	3	39
**DS4**	https://osf.io/dyx2q	12.5 × 12.5	800 × 800	150 × 150	3	39

Abbreviations as in Table A.

Table C: Stimulus set group for all experiments

	This paper		Kay et al 2013	
	OSF url		http://kendrickkay.net/socmodel/stimuli.mat	
	Number	Description	Number	Description
DS1	1 2 3 4 5	SNAKES (density)		
	47 48 3 49 50	SNAKES (contrast)		
	6 7 8 9 10	GRATINGS (density)		
	35 36 8 37 38	GRATINGS (contrast)		
	11 12 13 14 15	NOISE BARS (density)		
	16 17 18 19 20 21	WAVES (density)		
	8 22 23 24	GRATINGS (orientation)		
	13 25 26 27	NOISE BARS (orientation)		
	20 28 29 30	WAVES (orientation)		
	31 32 33 34	GRATINGS (spacing)		
	39 40 13 41 42	NOISE BARS (contrast)		
	43 44 18 45 46	WAVES (contrast)	Not part of Kay et al.	
DS2	1 2 3 4 5	SNAKES (density)		
	45 46 2 47 48	SNAKES (contrast)		
	6 7 8 9 10	GRATINGS (density)		
	33 34 7 35 36	GRATINGS (contrast)		
	11 12 13 14 15	NOISE BARS (density)		
	16 17 18 19	WAVES (density)	Not part of Kay et al.	

	8 20 21 22	GRATINGS (orientation)		
	13 23 24 25	NOISE BARS (orientation)		
	18 26 27 28	WAVES (orientation)		
	29 30 31 32	GRATINGS (cross)		
	37 38 13 39 40	NOISE BARS (contrast)		
	41 42 18 43 44	WAVES (contrast)		
DS3, DS4	36 37 38 39	SNAKES (density)	181-184	SEPARATION
	21 22 23 24 25 26 27 28 29 30	SNAKES (contrast)	159-168	CONTRAST
	31 32 33 34 35	GRATINGS (density)	176-180	not used
	9 10 11 12 1	GRATINGS (contrast)	147-150	GRATING
	1 2 3 4 5 6 7 8	GRATINGS (orientation)	139-146	ORIENTATION
	13 14 15 16	PLAID (contrast)	151-154	PLAID
	17 18 19 20	CIRCULAR (contrast)	155-158	CIRCULAR
	NA	not used	70-138	SPACE
	NA	not used	169-175	not used (objects/scenes)
	NA	not used	185-225	not used (mixtures)

Abbreviations as in Table A.

Fig A: Spectral power of snakes and gratings, data set 2

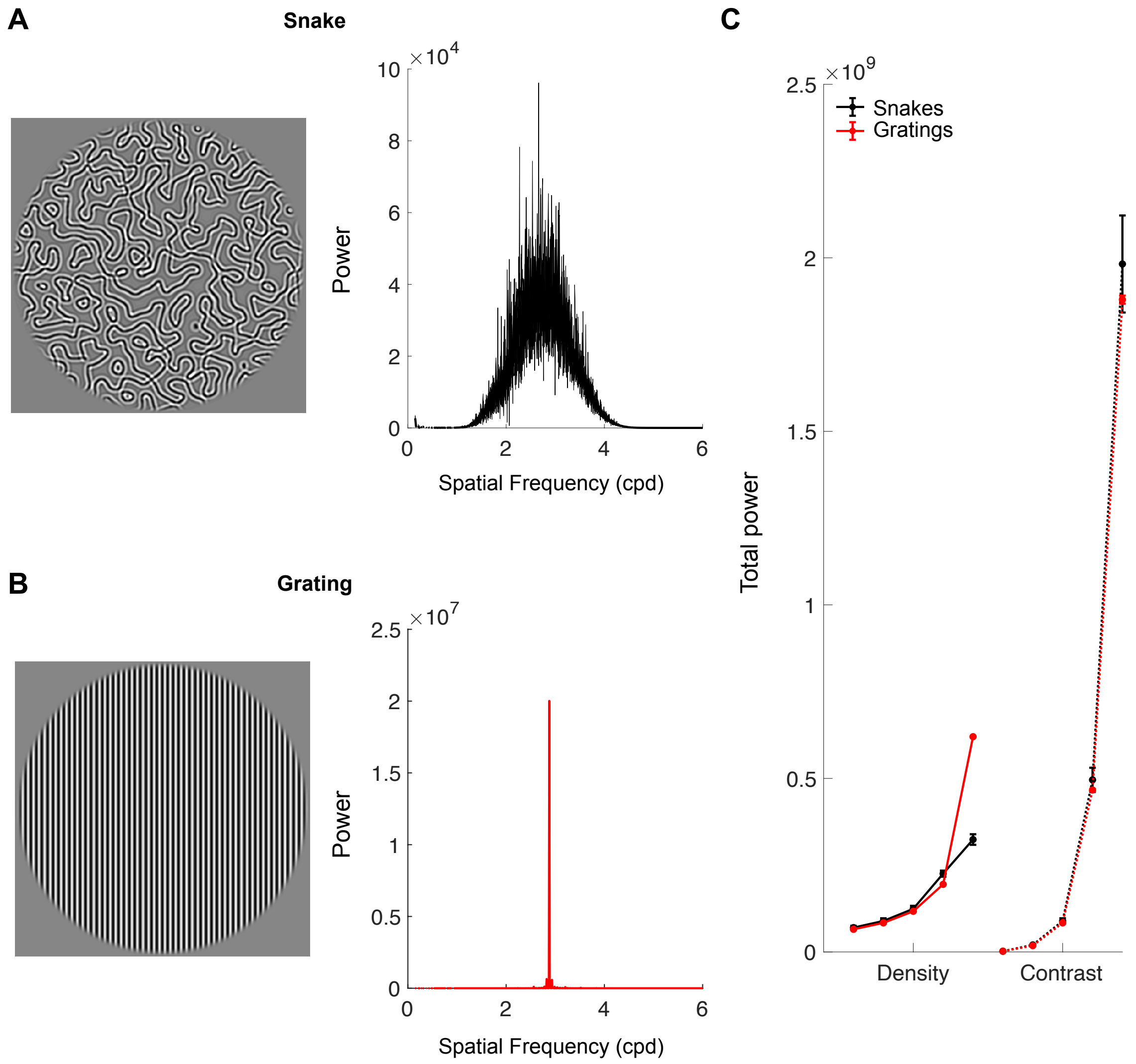

Supplement: S1 Appendix — (PDF) [file pcbi.1011704.s001.pdf]
