# Supplementary material for: Normalization by orientation-tuned surround in human V1-V3
Source: PLoS Comput Biol. 2023 Dec 27;19(12):e1011704. doi: 10.1371/journal.pcbi.1011704 (PMC10793941; doi:10.1371/journal.pcbi.1011704)

Stimuli images for data set 2

See Table B in S1 Data Set and Stimulus Properties for links to stimuli.

Snakes (Density)

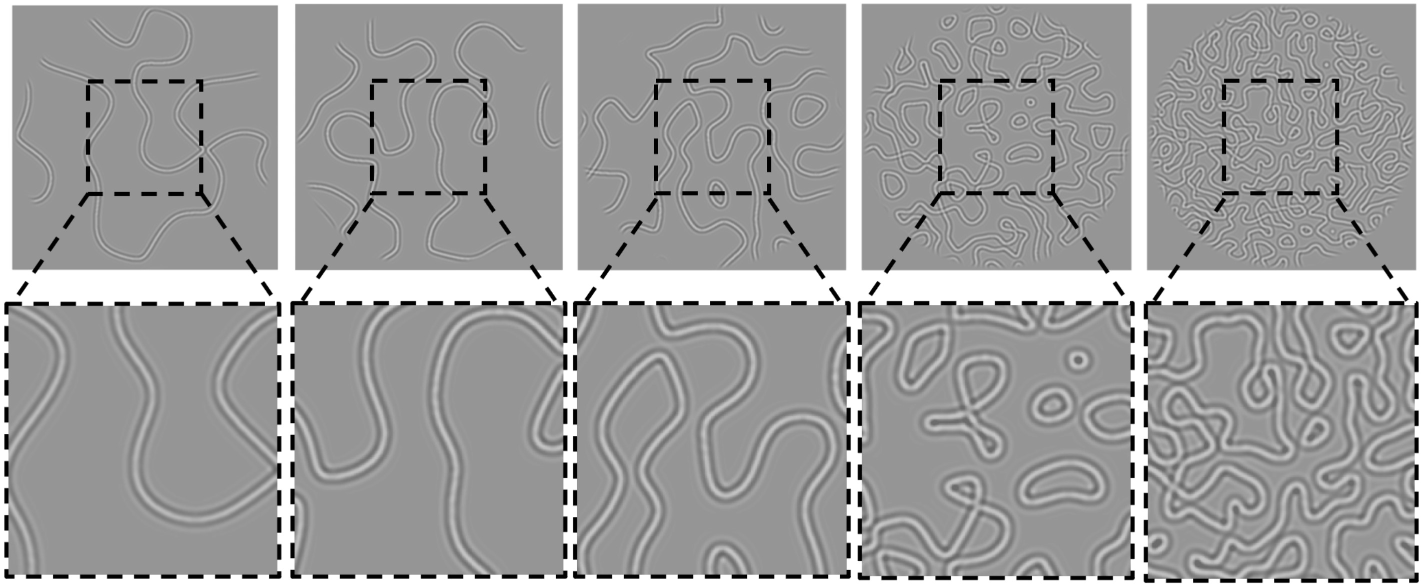

Gratings (Density)

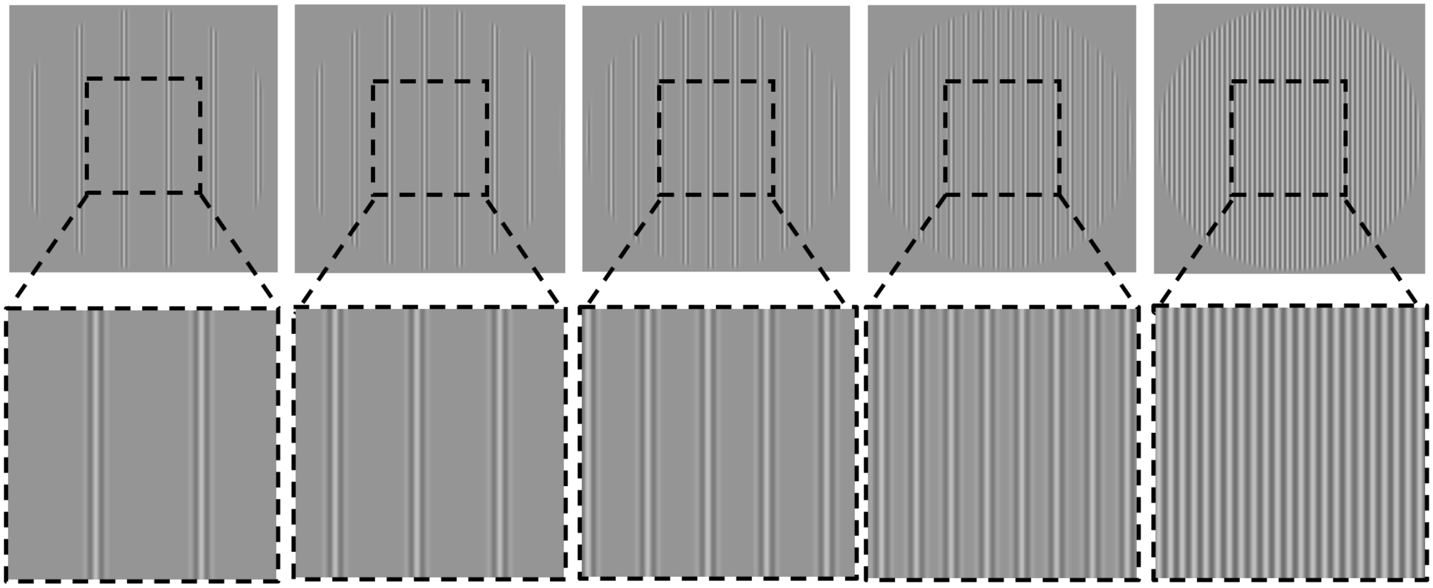

Noise Bars (Density)

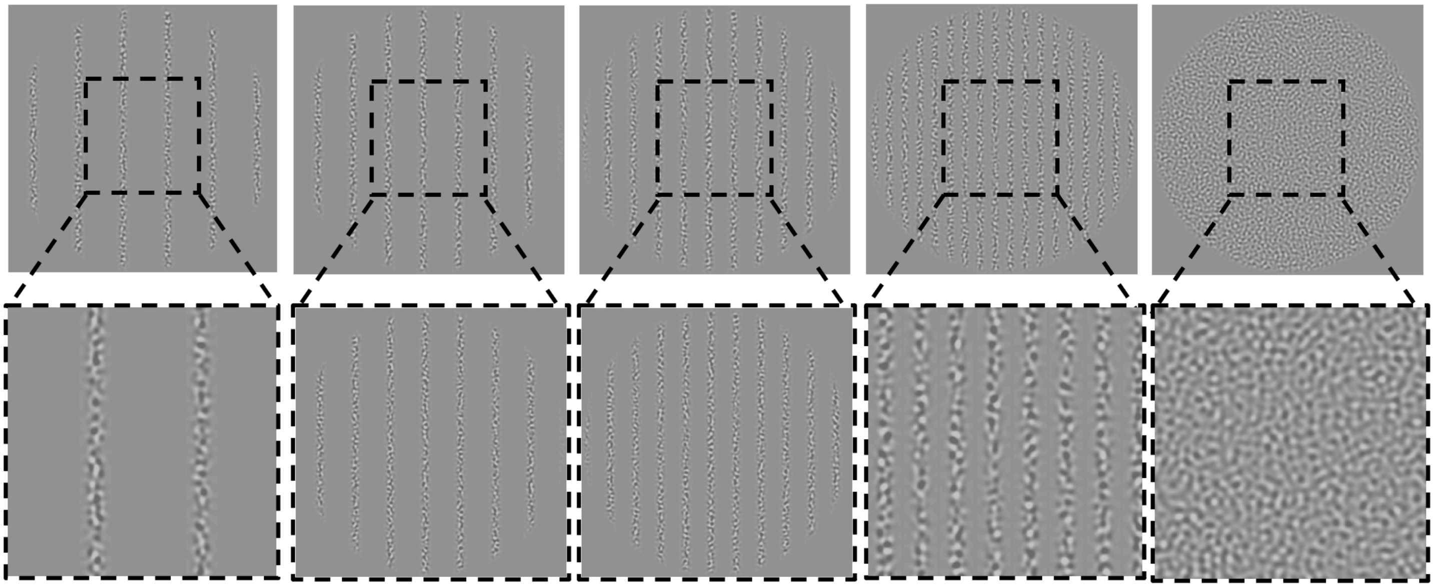

Waves (Density)

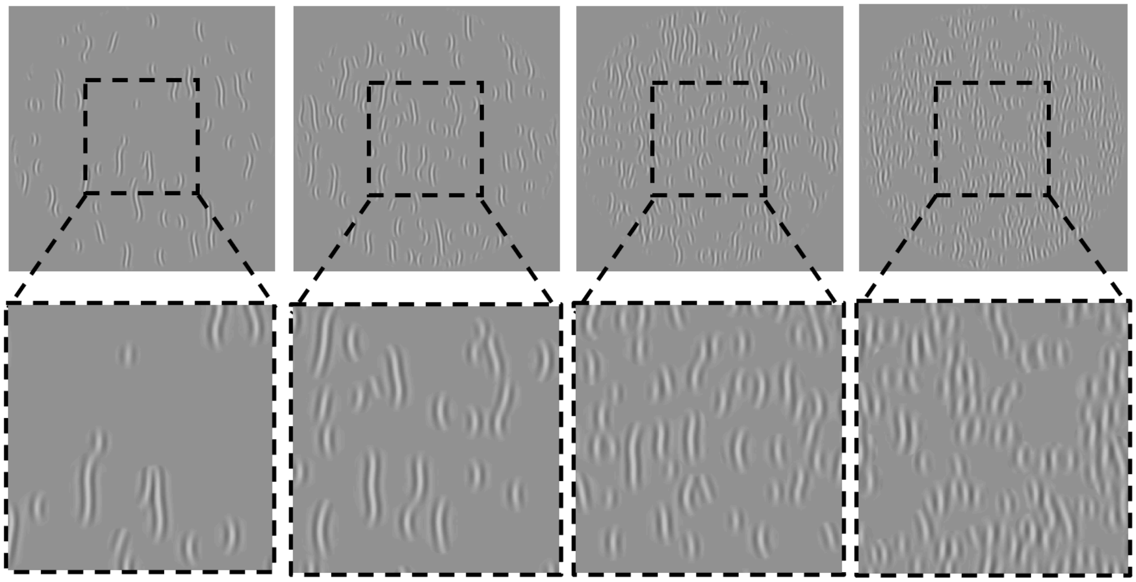

Gratings (Orientation)

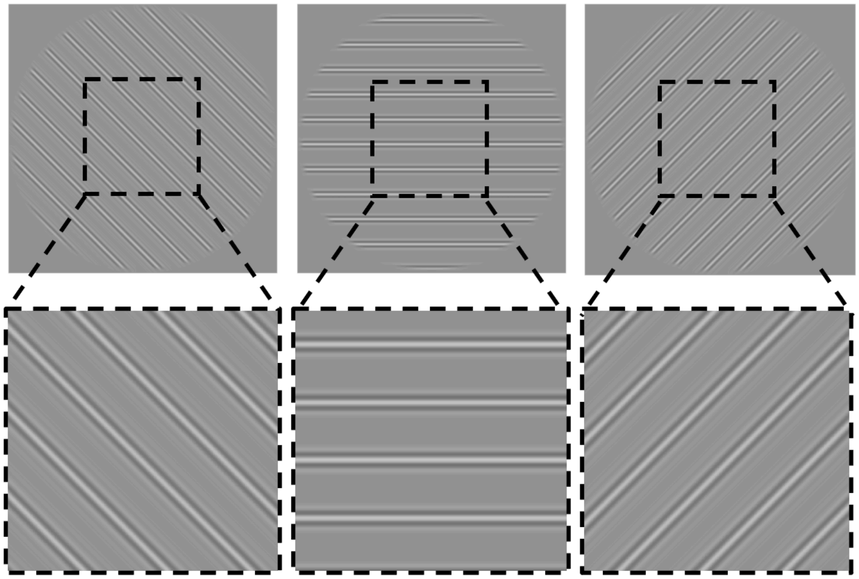

Noise Bars (Orientation)

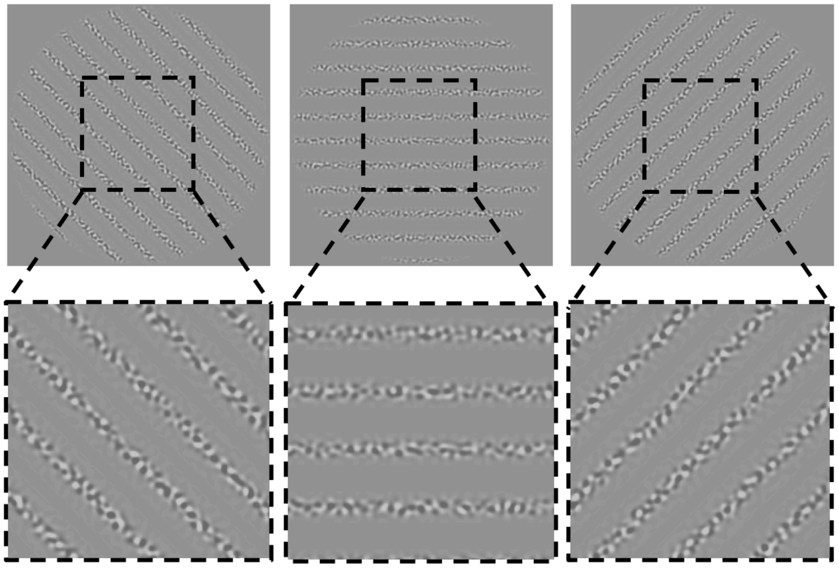

Waves (Orientation)

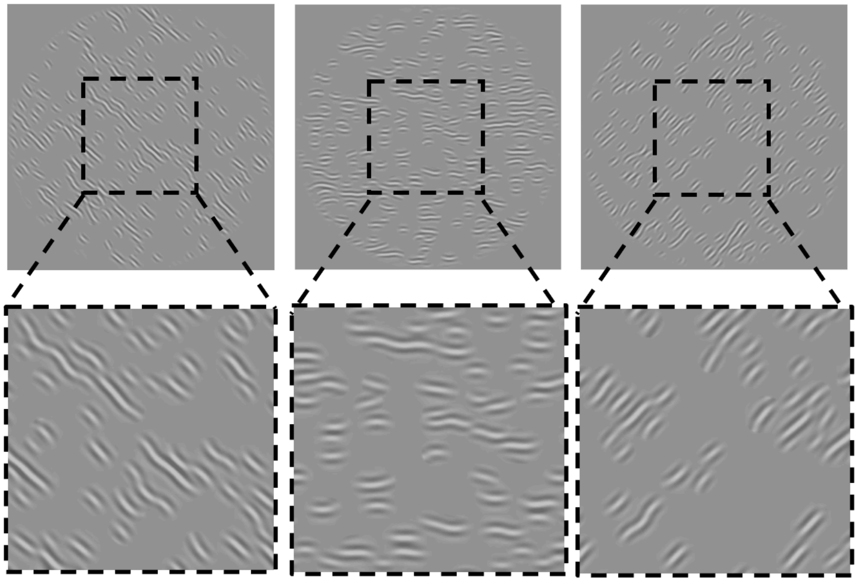

Gratings (Cross)

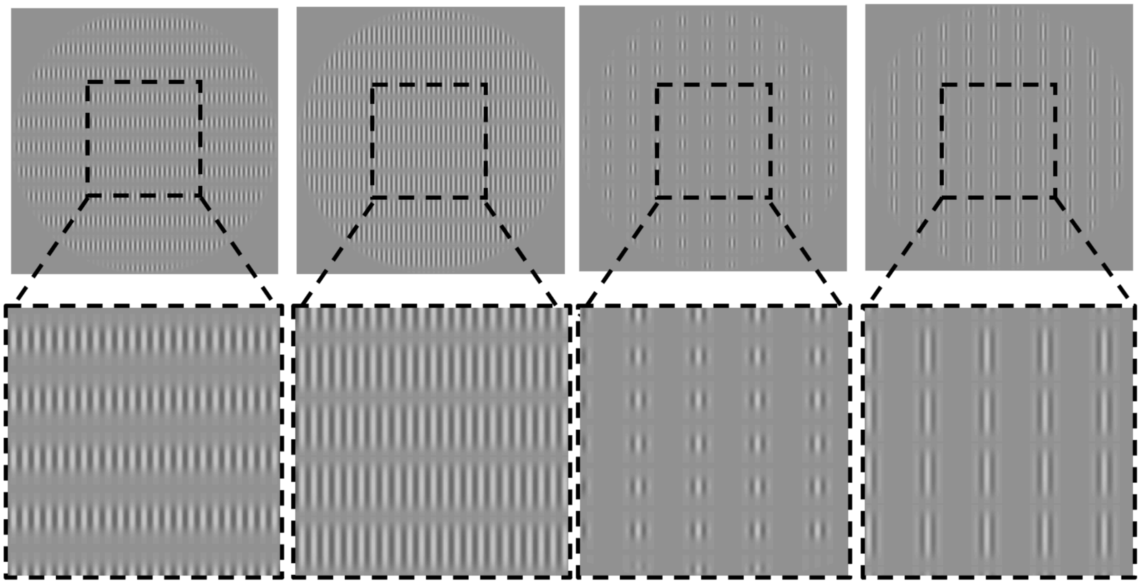

Gratings (Contrast)

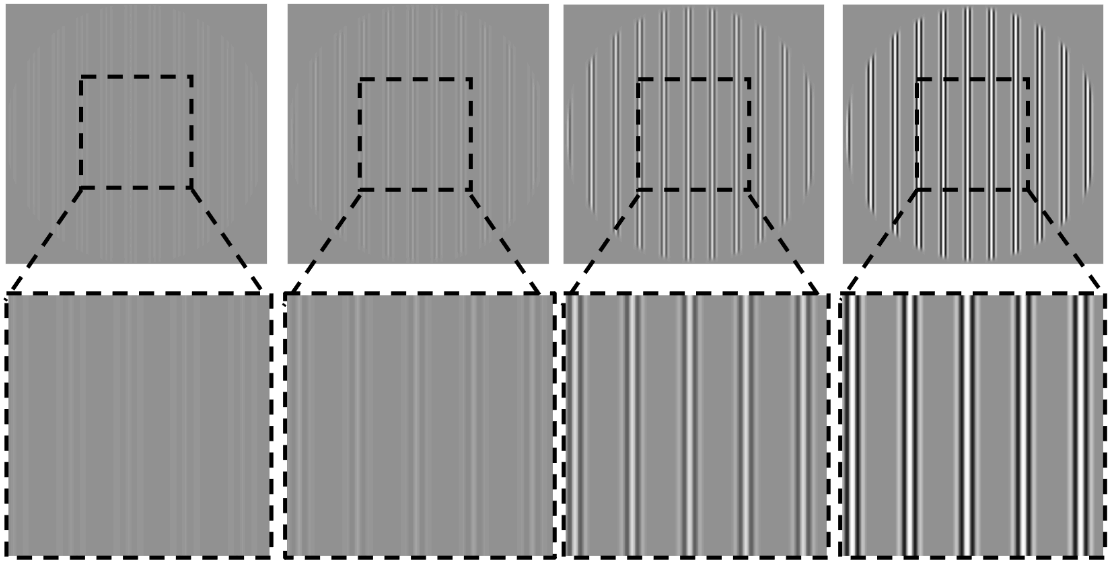

Noise Bars (Contrast)

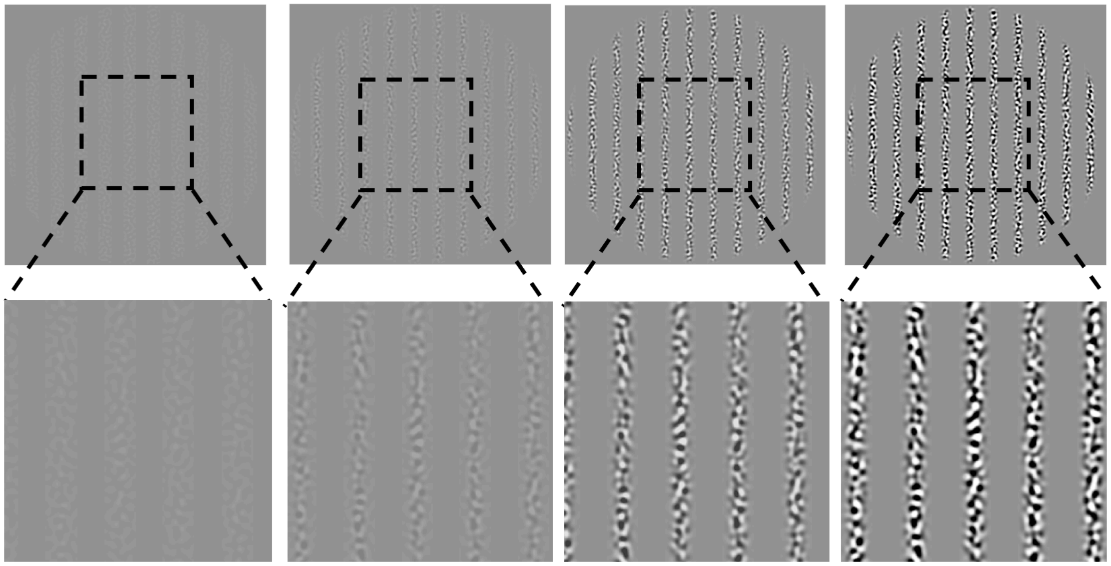

Waves (Contrast)

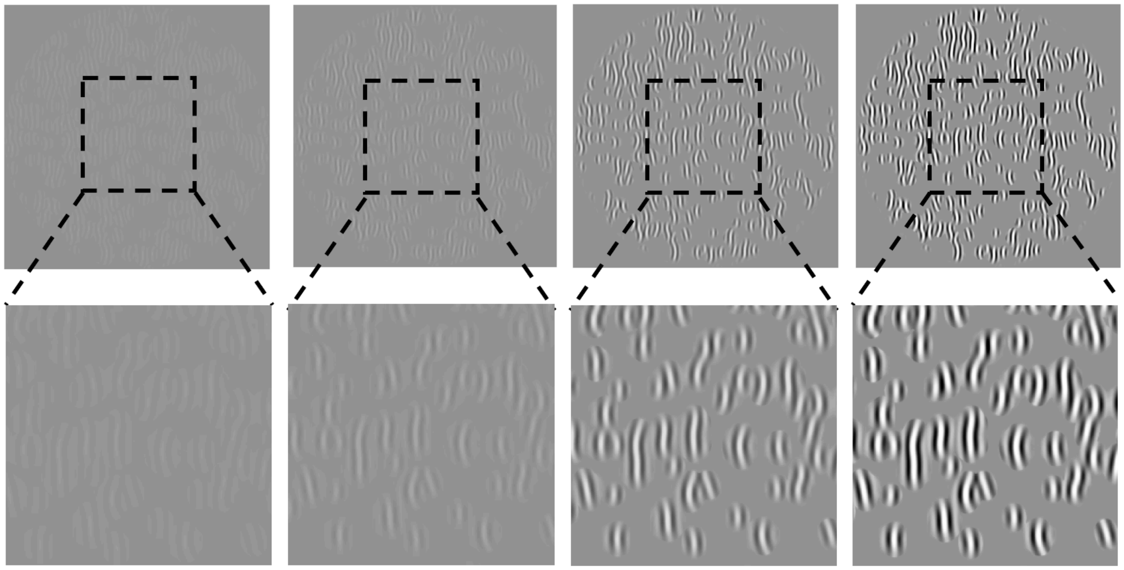

Snakes (Contrast)

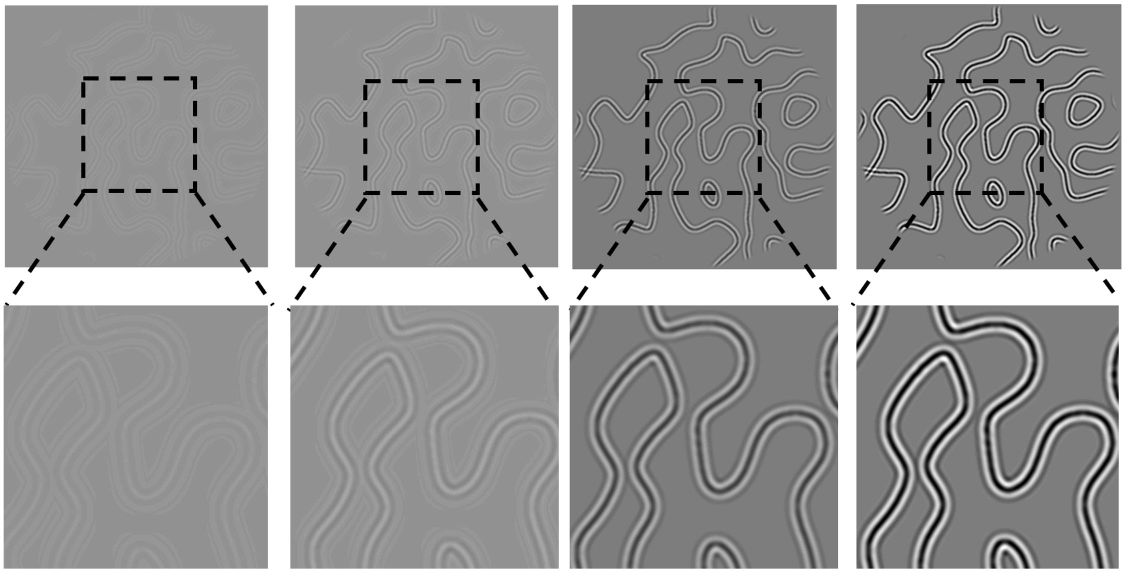

Supplement: S2 Appendix — (PDF) [file pcbi.1011704.s002.pdf]
