# Supplementary material for: Normalization by orientation-tuned surround in human V1-V3
Source: PLoS Comput Biol. 2023 Dec 27;19(12):e1011704. doi: 10.1371/journal.pcbi.1011704 (PMC10793941; doi:10.1371/journal.pcbi.1011704)
Supplement: S3 Appendix — (PDF) [file pcbi.1011704.s003.pdf]

Model Variance Explained

Table A: Cross-validated variance explained for 4 models on target stimuli

A. Target stimuli												
**Model** (free params)	**V1**				**V2**				**V3**			
	**DS1**	**DS2**	**DS3**	**DS4**	**DS1**	**DS2**	**DS3**	**DS4**	**DS1**	**DS2**	**DS3**	**DS4**
Contrast (2)	-0.031	0.458	-0.111	0.037	-0.133	0.176	-0.179	-0.139	-0.147	-0.042	-0.167	-0.158
DN (3)	-0.120	0.369	-0.158	0.277	-0.175	0.129	-0.180	0.087	-0.201	-0.024	-0.179	-0.008
OTN (3)	**0.298**	**0.796**	**0.664**	**0.700**	0.748	**0.796**	**0.776**	**0.707**	**0.897**	**0.798**	**0.708**	**0.742**
NOA (3)	0.026	0.768	0.504	0.446	**0.768**	0.692	0.583	0.490	0.794	0.780	0.496	0.499

Table A. Cross-validated variance explained for 4 models on *target stimuli*. The table shows the cross validated coefficient of determination (R^2) for four data sets in three visual areas. The number of fitted model parameters (degrees of freedom) is indicated in parentheses for each model type (column 1). The number of stimuli comprising the target set are 18 for data set 1 (DS1) and data set 2 (DS2) and 17 for data set 3 (DS3), data set (DS4). The bold-faced font indicates the model with the highest variance explained in that column.

Table B: Cross-validated variance explained for 4 models on the full stimulus set

B. All stimuli												
**Model** (free params)	**V1**				**V2**				**V3**			
	**DS1**	**DS2**	**DS3**	**DS4**	**DS1**	**DS2**	**DS3**	**DS4**	**DS1**	**DS2**	**DS3**	**DS4**
Contrast (2)	0.146	0.528	0.362	0.437	0.060	0.376	0.114	0.003	0.057	0.250	0.121	0.038
DN (3)	0.240	0.524	0.428	0.508	0.162	0.425	0.328	0.142	0.113	0.339	0.278	0.172
OTN (3)	**0.548**	**0.769**	**0.657**	**0.673**	**0.638**	**0.696**	**0.731**	**0.705**	**0.637**	**0.627**	**0.669**	**0.670**
NOA (3)	0.424	0.652	0.554	0.546	0.436	0.553	0.607	0.583	0.520	0.487	0.517	0.563

Table B. Cross-validated variance explained for 4 models on the full stimulus set. The table is organized in the same way as Table 1, differing only in the number of stimuli used to fit the models (50 for data set 1; 48 for data set 2; 39 for data set 3, data set 4).
