# Supplementary material for: Normalization by orientation-tuned surround in human V1-V3
Source: PLoS Comput Biol. 2023 Dec 27;19(12):e1011704. doi: 10.1371/journal.pcbi.1011704 (PMC10793941; doi:10.1371/journal.pcbi.1011704)
Supplement: S4 Appendix — (PDF) [file pcbi.1011704.s004.pdf]

Model Parameter Estimates

Table A: Cross-validated fitted parameters for 4 models on the target stimulus set

A. Target stimuli													
		**V1**				**V2**				**V3**			
		**DS1**	**DS2**	**DS3**	**DS4**	**DS1**	**DS2**	**DS3**	**DS4**	**DS1**	**DS2**	**DS3**	**DS4**
CE	g	0.916	2.590	1.430	2.040	0.928	1.250	1.530	1.710	0.953	1.140	1.560	2.080
	α	0.170	0.143	0.084	0.130	0.086	0.096	0.044	0.091	0.060	0.069	0.046	0.082
DN	σ	807.0	3445.0	0.033	0.011	192.0	0.135	0.009	0.007	621.0	0.011	0.012	0.008
	g	1.240	4.040	9.350	13.200	1.050	1.190	9.140	11.900	1.070	1.160	4.930	12.700
	α	0.282	0.172	0.950	1.000	0.136	0.137	0.865	0.999	0.088	0.165	0.570	0.915
OTN	σ	0.003	0.050	0.034	0.029	0.001	0.009	0.008	0.007	0.000	0.003	0.006	0.006
	g	7.460	3.950	5.570	7.310	7.410	2.190	4.700	7.880	7.090	2.540	4.390	8.610
	α	0.965	0.322	0.510	0.529	0.807	0.339	0.415	0.608	0.747	0.394	0.389	0.565
NOA	σ	0.001	0.005	0.002	0.001	0.000	0.001	0.000	0.000	0.000	0.000	0.000	0.000
	g	0.333	1.540	0.980	1.210	0.497	0.818	1.180	1.050	0.601	0.781	1.200	1.340
	α	0.662	0.246	0.260	0.262	0.530	0.230	0.197	0.273	0.452	0.257	0.181	0.256

Table A. Cross-validated fitted parameters for 4 models on the target stimulus set. The table is organized in a similar way with the R^2 table (18 for data set 1, data set 2; 17 for data set 3, data set 4). CE = contrast energy; DN = untuned normalization; OTN = orientation-tuned normalization; NOA = normalization by anisotropy.

Table B: Cross-validated fitted parameters for 4 models on the full stimulus set

B. All stimuli													
		**V1**				**V2**				**V3**			
		**DS1**	**DS2**	**DS3**	**DS4**	**DS1**	**DS2**	**DS3**	**DS4**	**DS1**	**DS2**	**DS3**	**DS4**
CE	g	1.140	2.980	1.450	1.890	1.110	1.590	1.370	1.270	1.170	1.430	1.420	1.630
	α	0.197	0.150	0.105	0.119	0.097	0.121	0.050	0.048	0.079	0.100	0.049	0.054
DN	σ	0.003	0.056	0.005	0.007	0.003	0.011	0.002	0.002	0.004	0.004	0.003	0.006
	g	3.880	2.800	9.590	12.700	1.640	1.690	5.170	7.540	1.320	1.670	3.360	4.440
	α	1.000	0.247	0.982	0.986	0.398	0.315	0.668	0.867	0.234	0.341	0.447	0.501
OTN	σ	0.009	0.059	0.123	0.128	0.002	0.035	0.017	0.009	0.002	0.017	0.014	0.011
	g	3.640	3.900	4.150	5.350	2.930	2.110	4.000	7.140	2.630	1.920	3.770	7.740
	α	0.709	0.301	0.359	0.365	0.505	0.280	0.364	0.588	0.417	0.271	0.340	0.533
NOA	σ	0.001	0.011	0.024	0.041	0.001	0.006	0.003	0.001	0.001	0.004	0.003	0.002
	g	0.387	1.780	1.370	1.830	0.649	1.010	1.280	1.120	0.762	0.952	1.310	1.440
	α	0.393	0.209	0.220	0.221	0.256	0.185	0.179	0.250	0.218	0.169	0.161	0.236

Table B. Cross-validated fitted parameters for 4 models on the full stimulus set. The table is organized in a similar way with the R^2 table (50 for data set 1; 48 for data set 2; 39 for data set 3, data set 4). Abbreviations as in table A.
