# Supplementary material for: Normalization by orientation-tuned surround in human V1-V3
Source: PLoS Comput Biol. 2023 Dec 27;19(12):e1011704. doi: 10.1371/journal.pcbi.1011704 (PMC10793941; doi:10.1371/journal.pcbi.1011704)

Plots of Model Fits

Fig A1: Responses and contrast energy model fits for target stimuli, all data sets

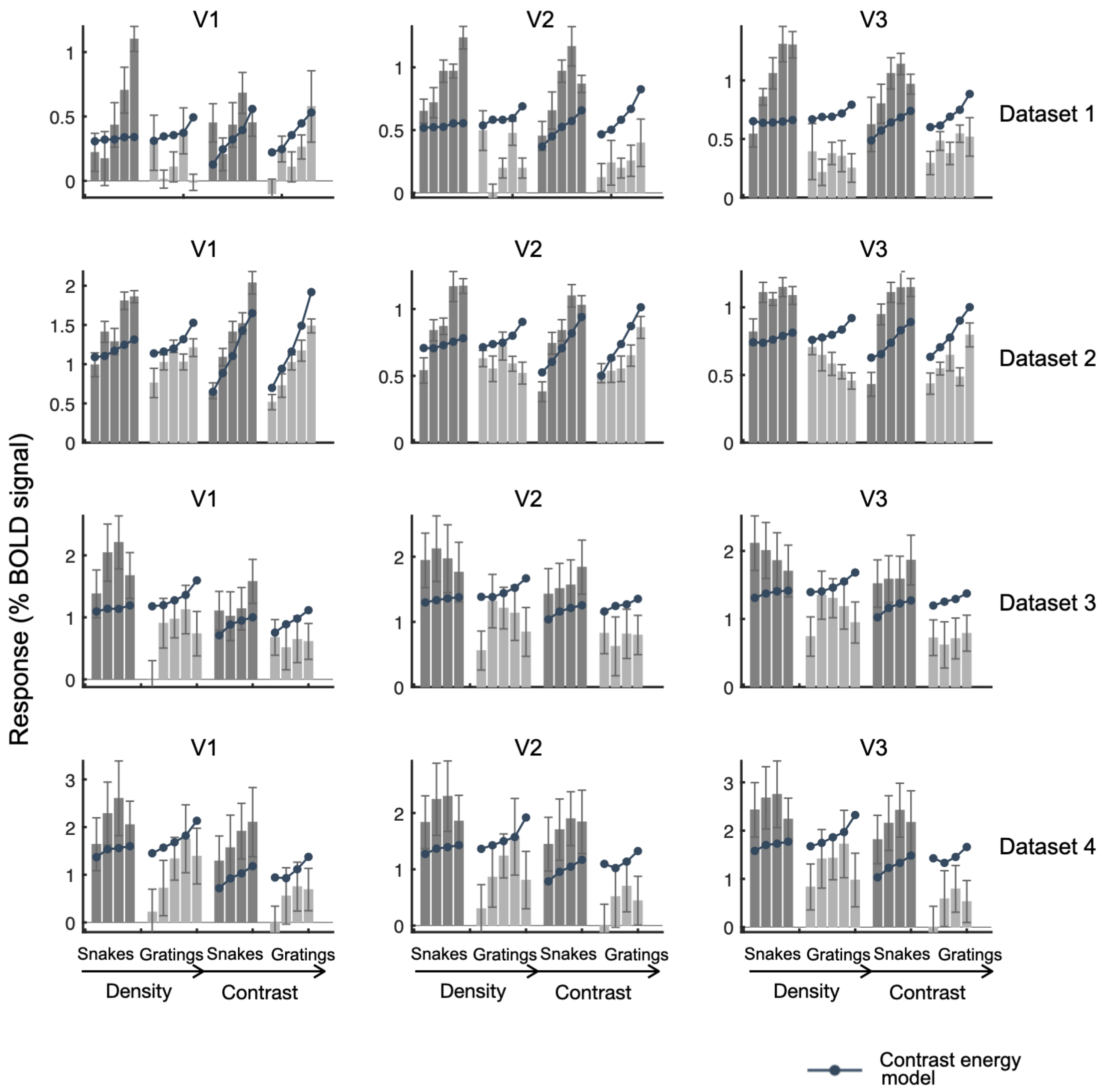

Fig A2: Responses and Untuned normalization fits for target stimuli, all data sets

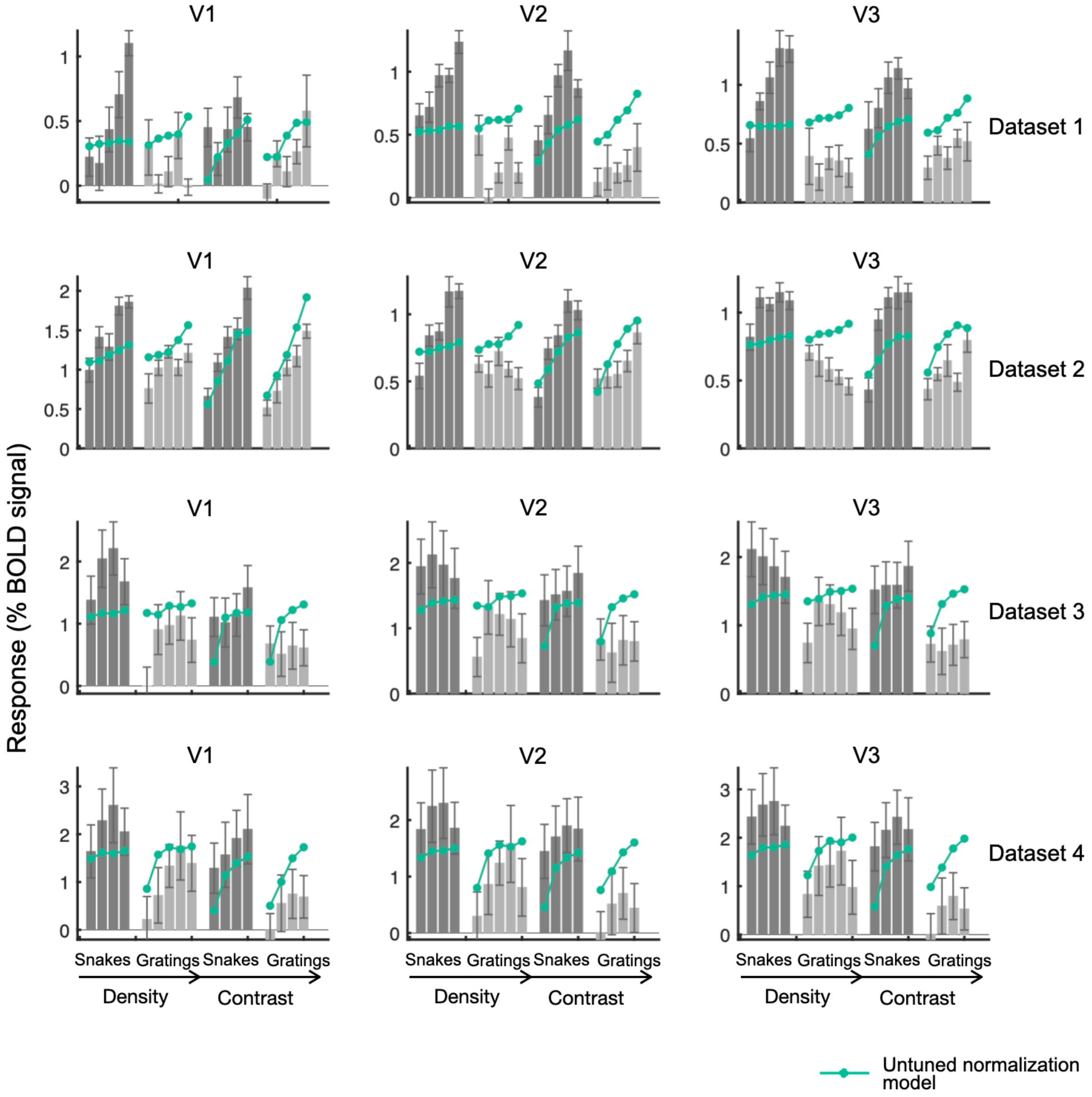

Fig A3: Responses and orientation -tuned normalization model fits for target stimuli, all data sets

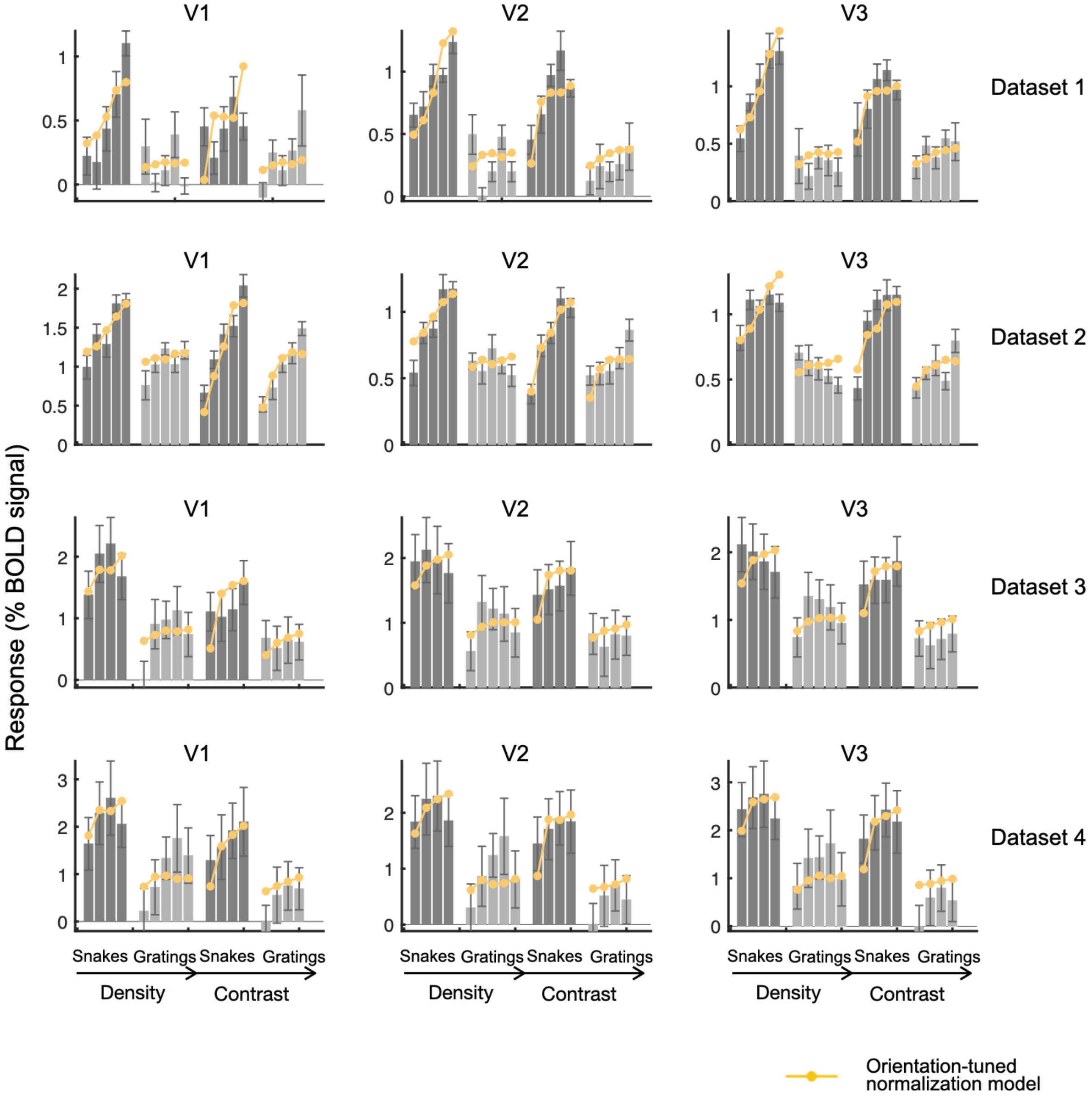

Fig A4: Responses and normalization by orientation anisotropy model fits for target stimuli, all data sets

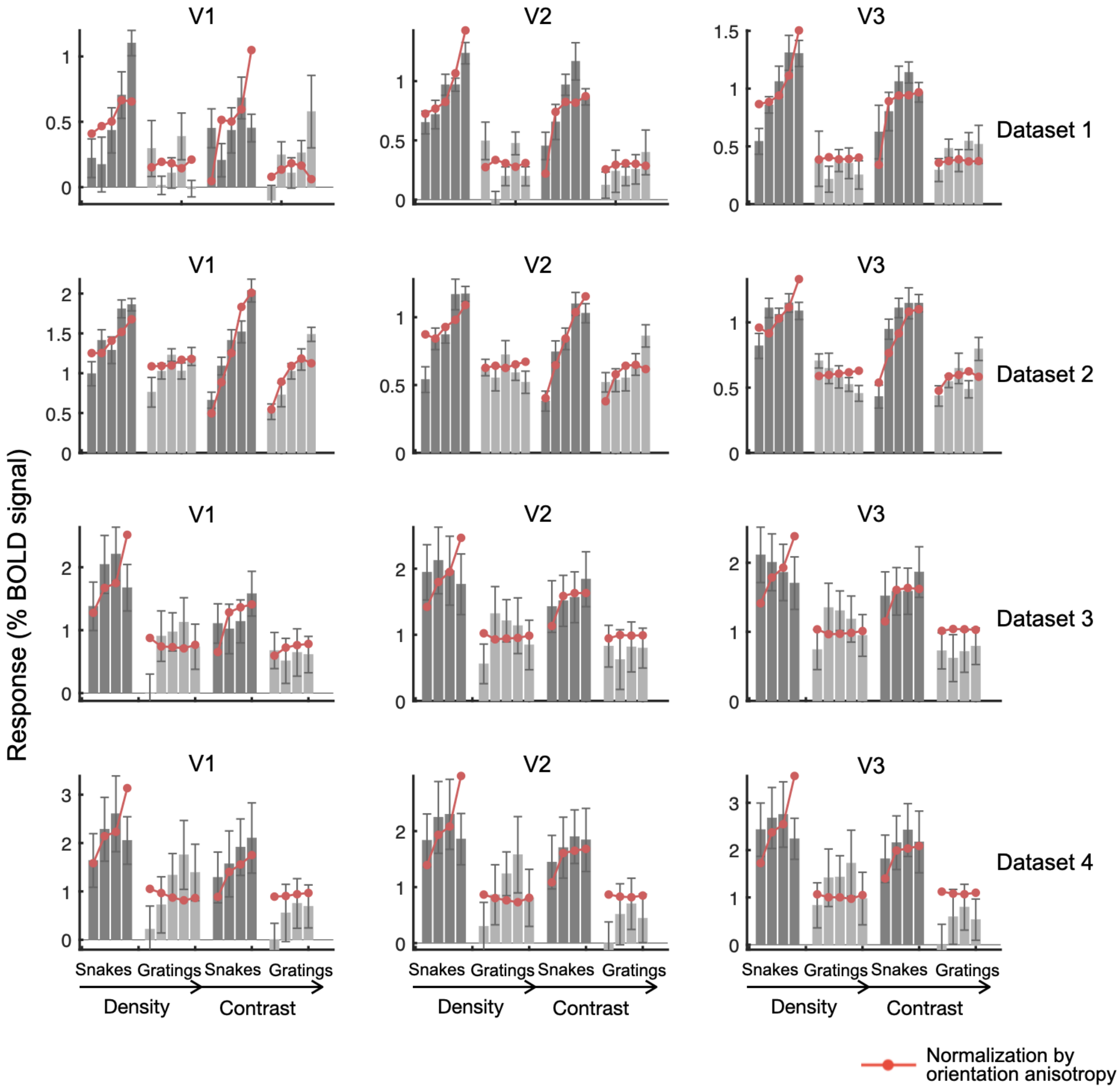

Fig B1: Responses and contrast energy model fits for all stimuli, data set 1

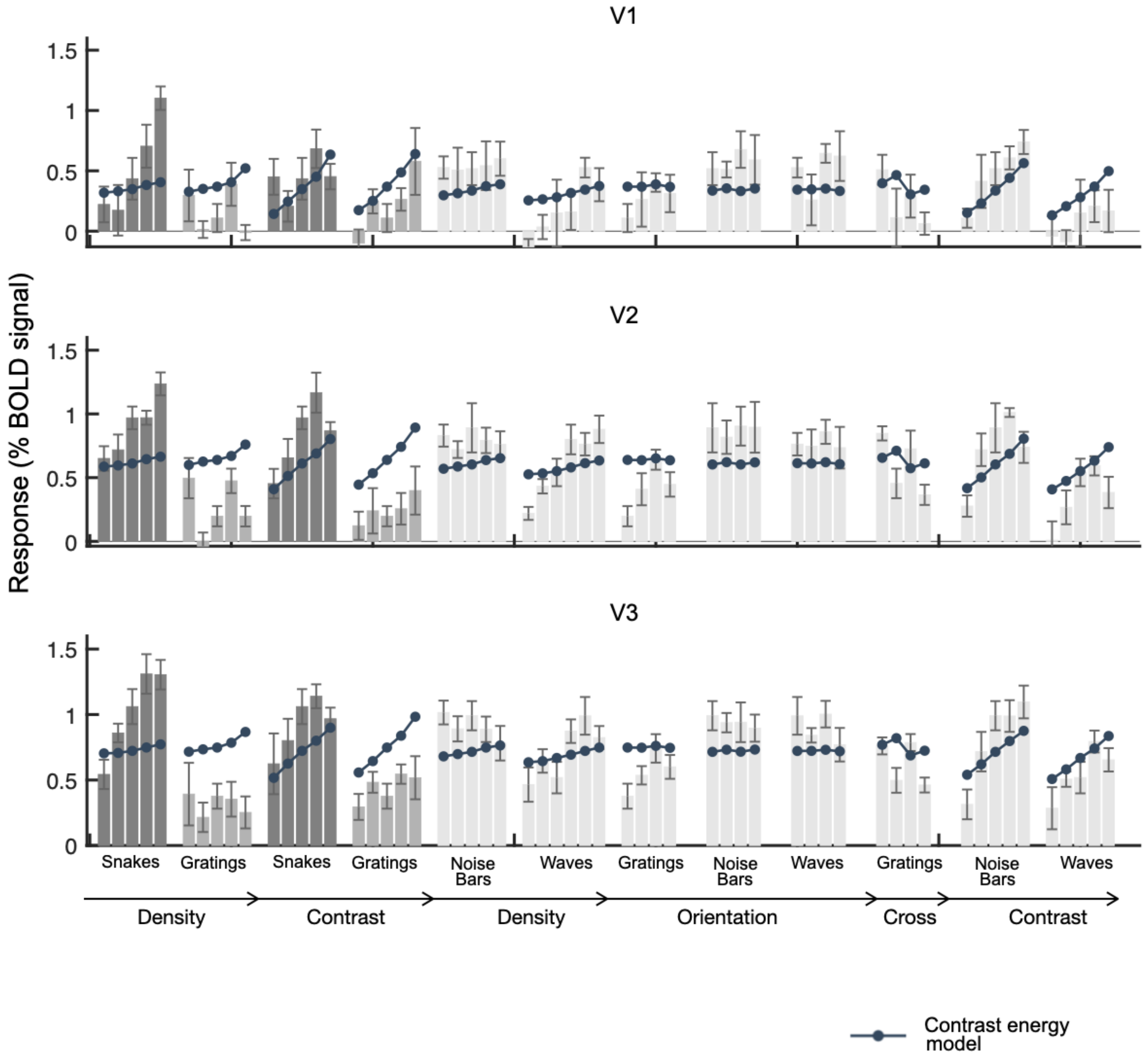

Fig B2: Responses and untuned normalization model fits for all stimuli, data set 1

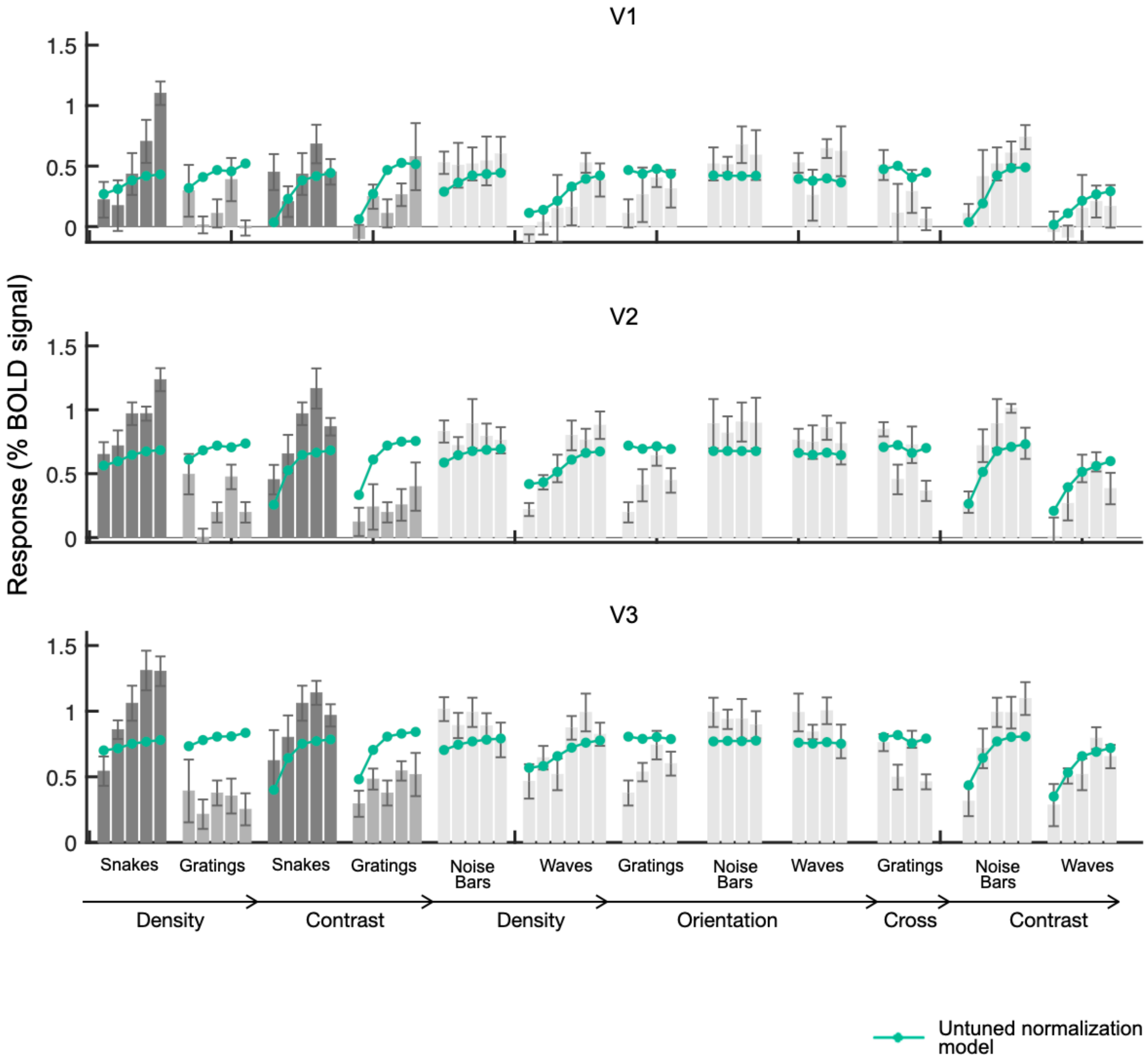

Fig B3: Responses and orientation-tuned normalization model fits for all stimuli, data set 1

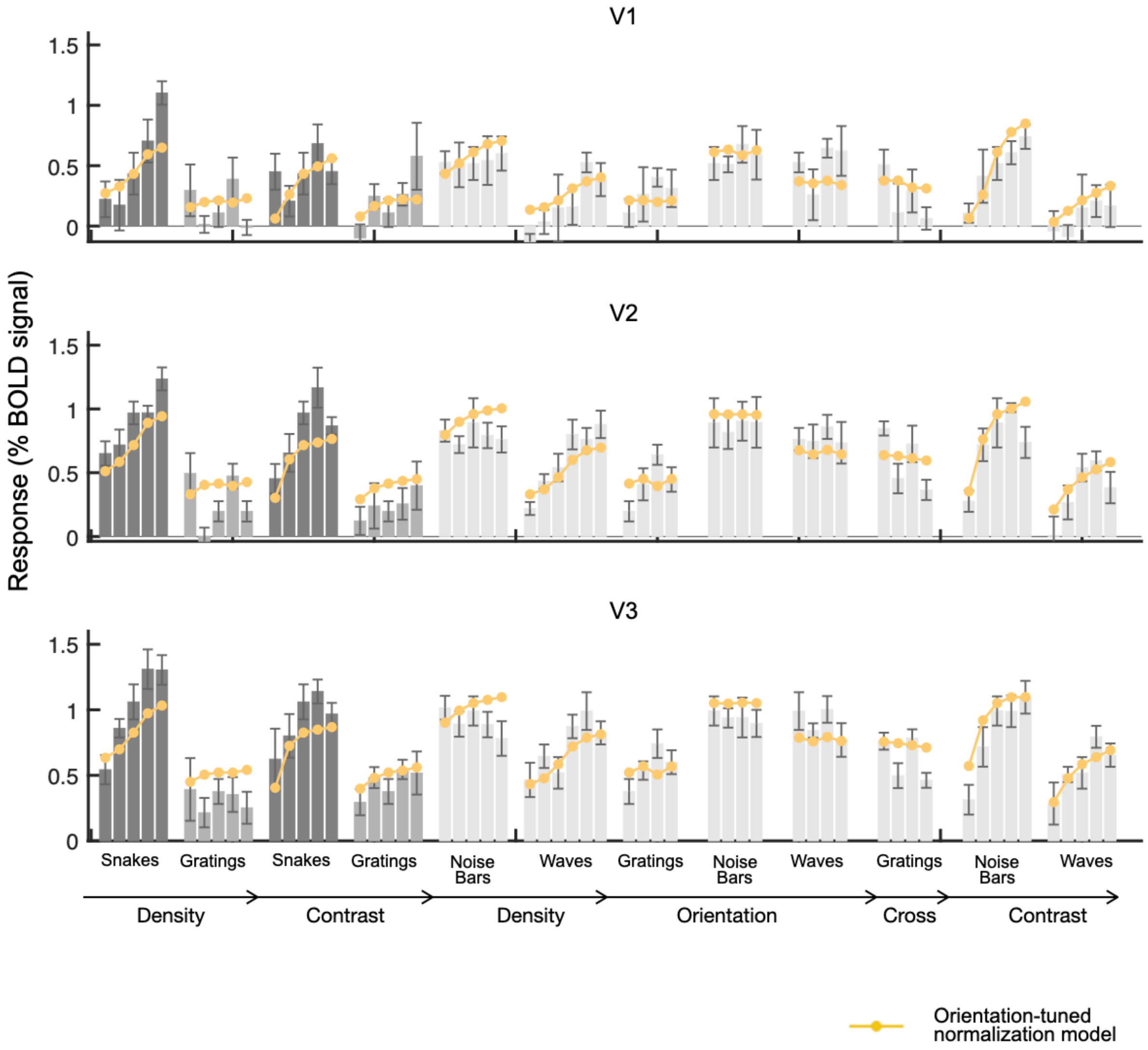

Fig B4: Responses and normalization by orientation anisotropy model fits for all stimuli, data set 1

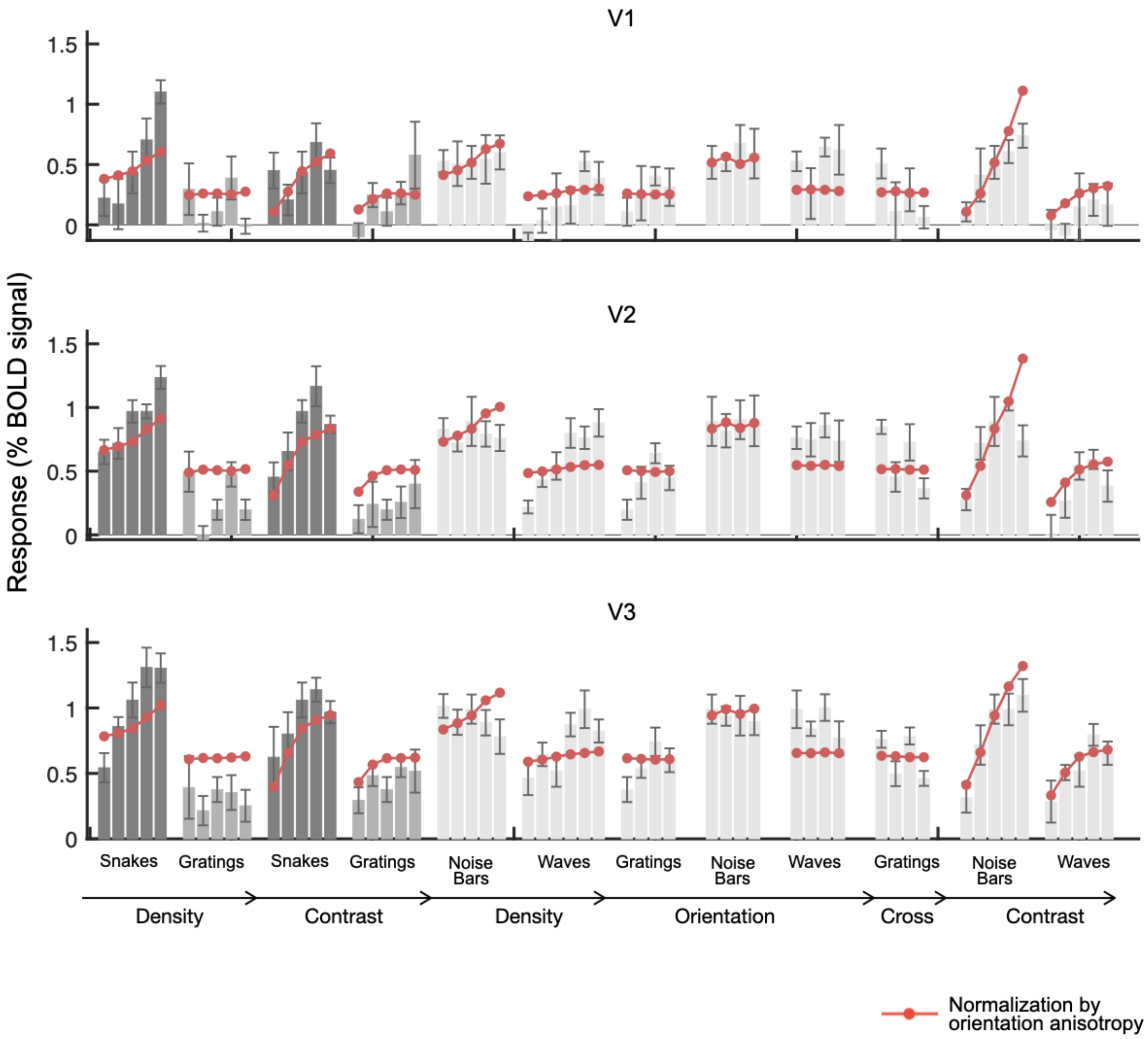

Fig C1: Responses and contrast energy model fits for all stimuli, data set 2

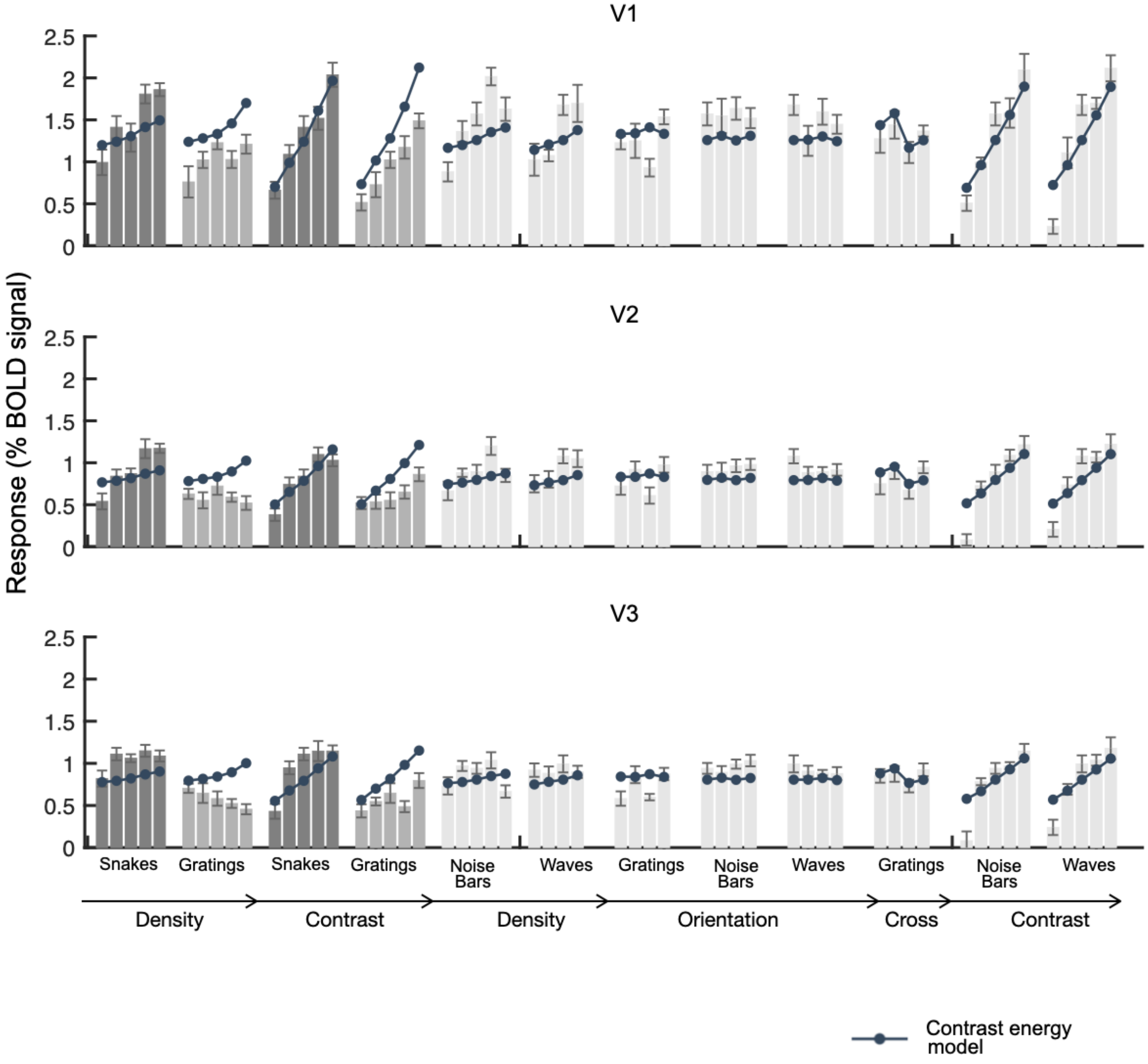

Fig C2: Responses and untuned normalization model fits for all stimuli, data set 2

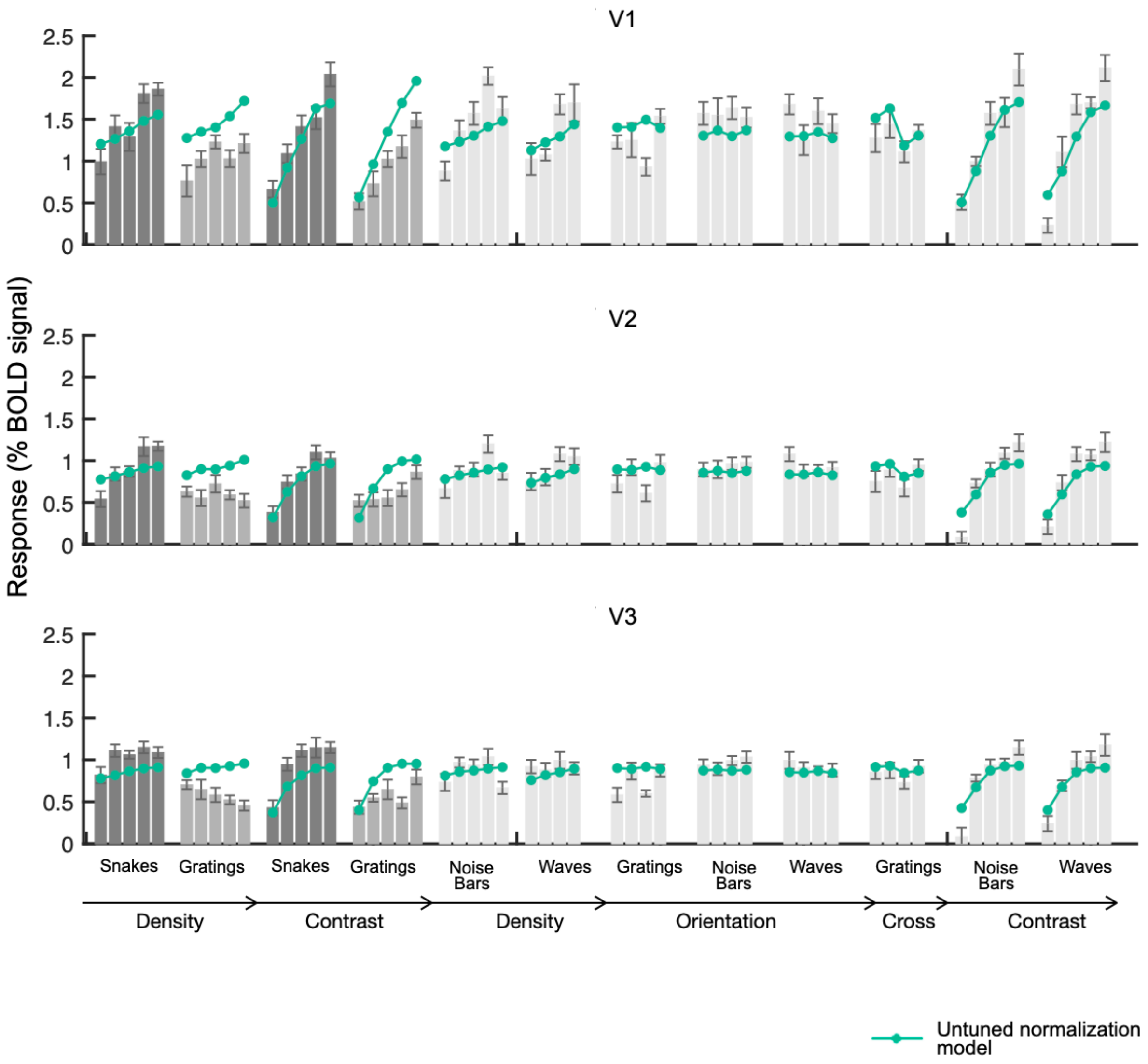

Fig C3: Responses and orientation-tuned normalization model fits for all stimuli, data set 2

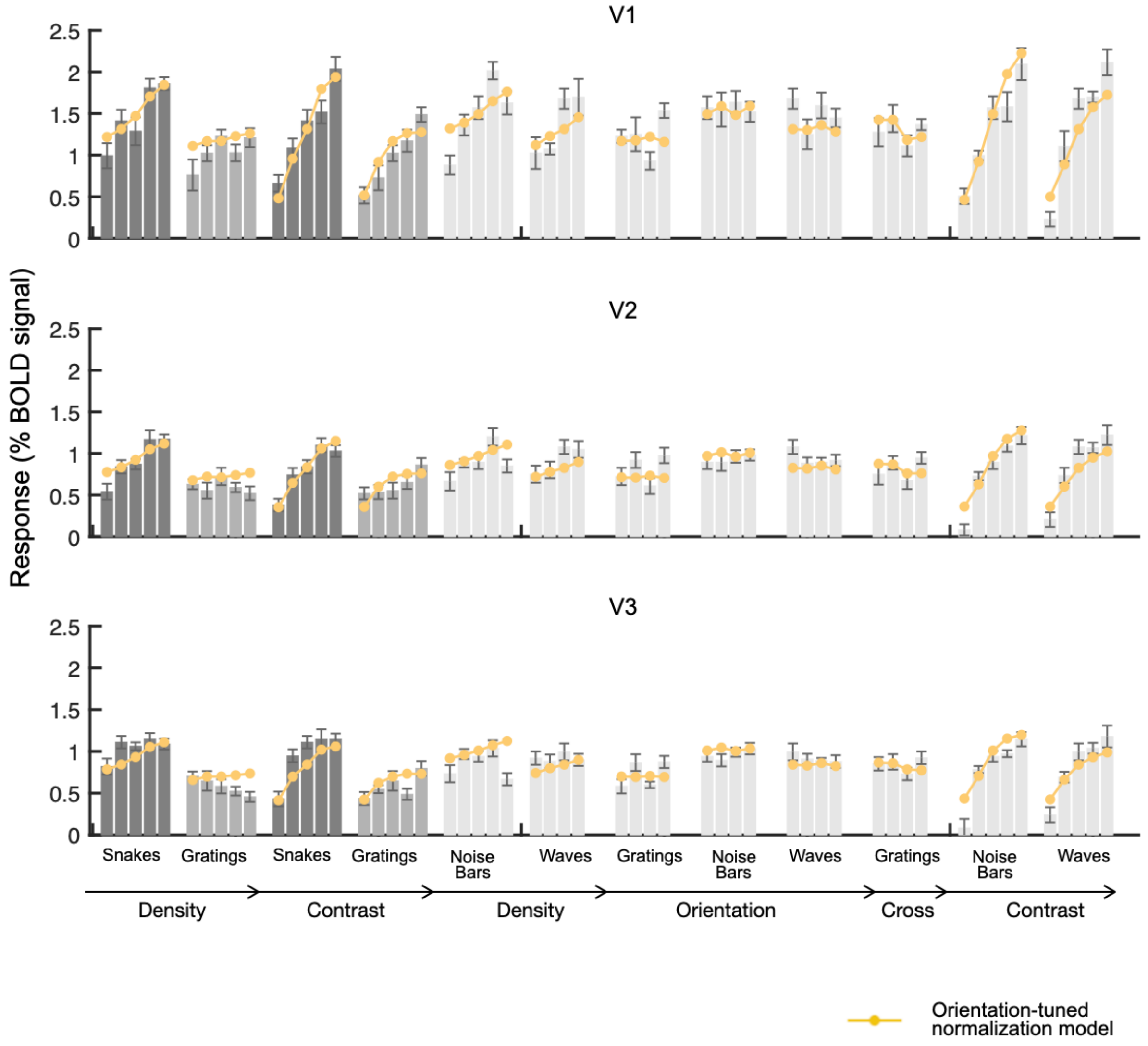

Fig C4: Responses and normalization by orientation anisotropy model fits for all stimuli, data set 2

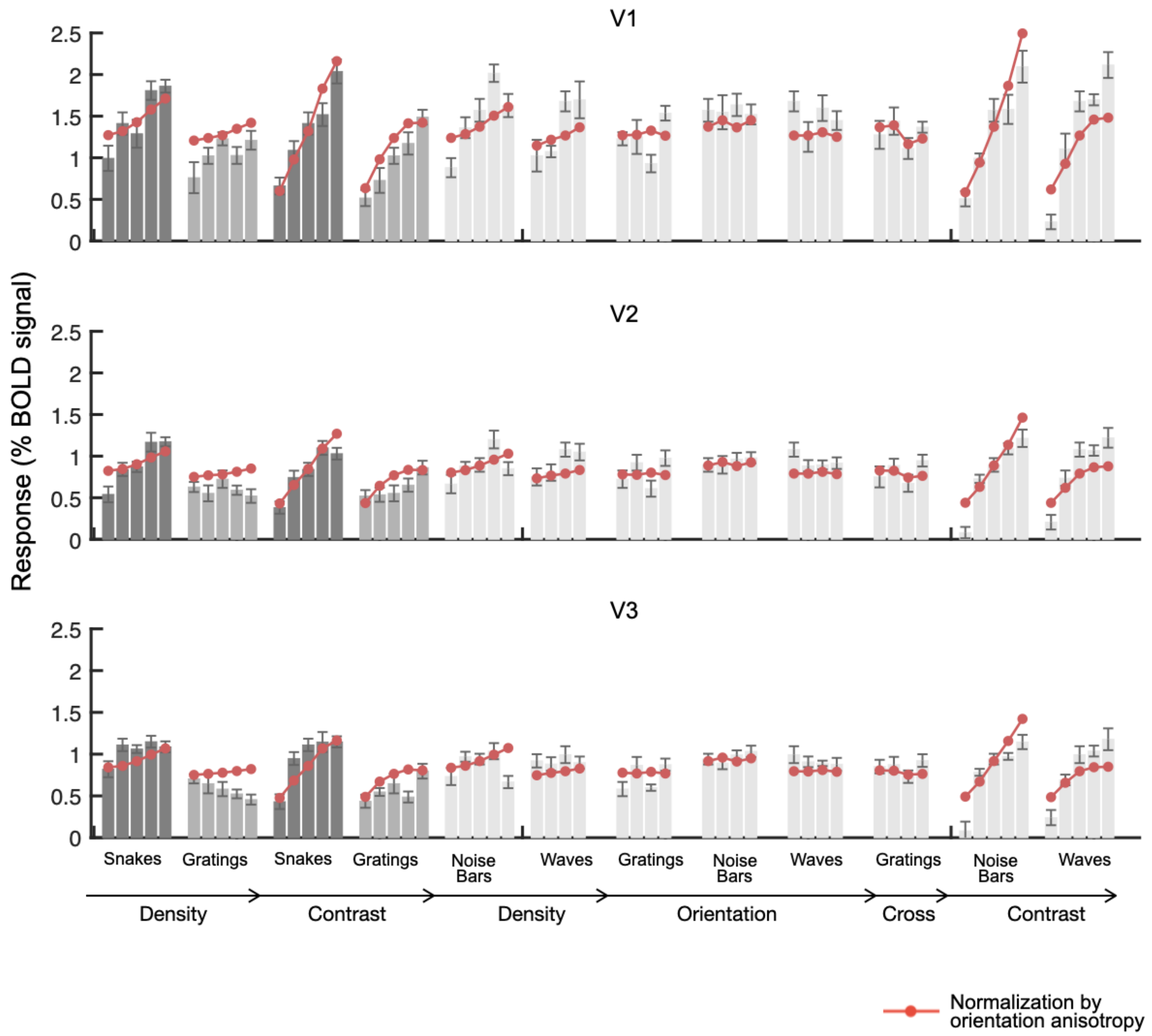

Fig D1: Responses and contrast energy model fits for all stimuli, data set 3

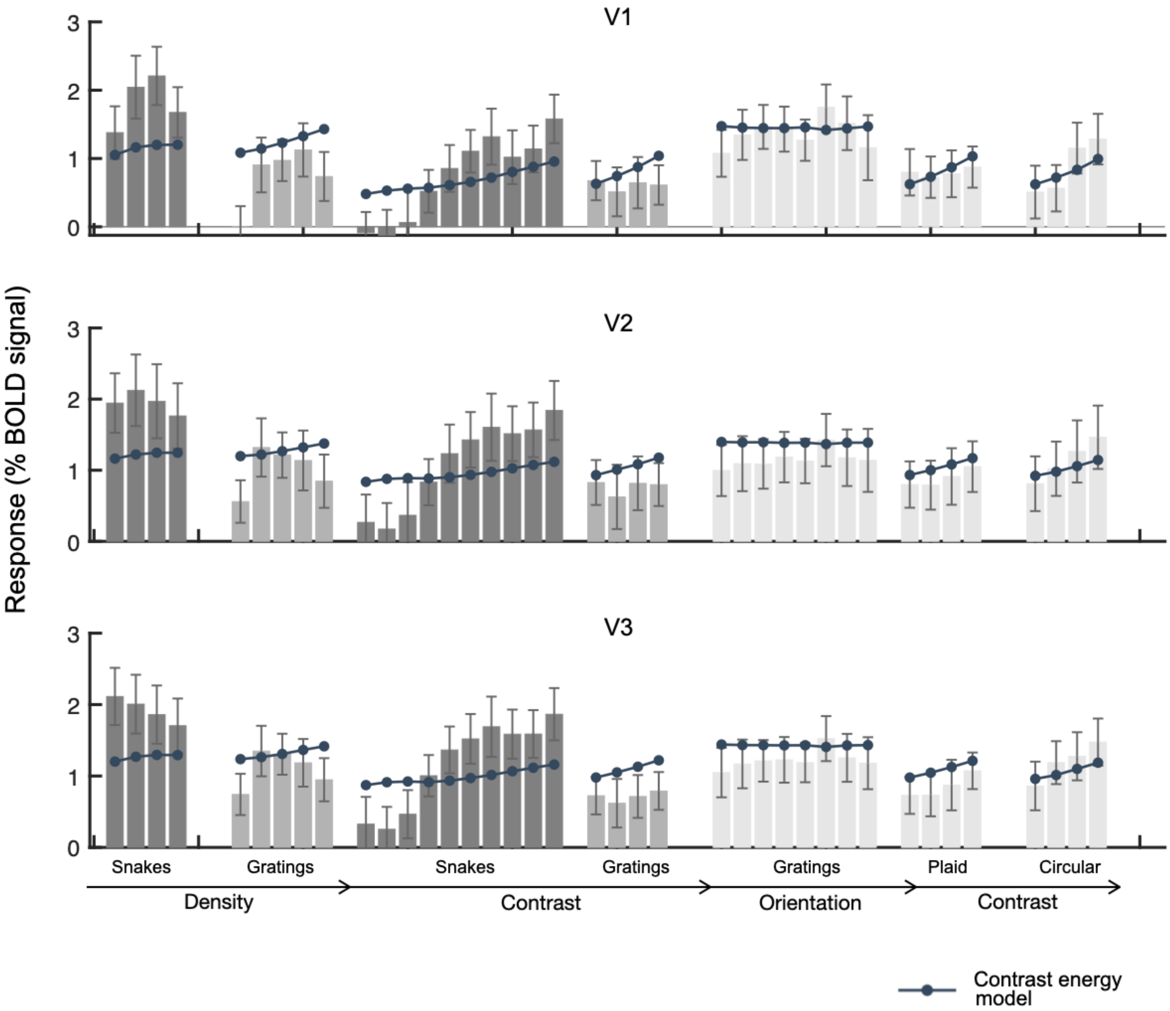

Fig D2: Responses and untuned normalization model fits for all stimuli, data set 3

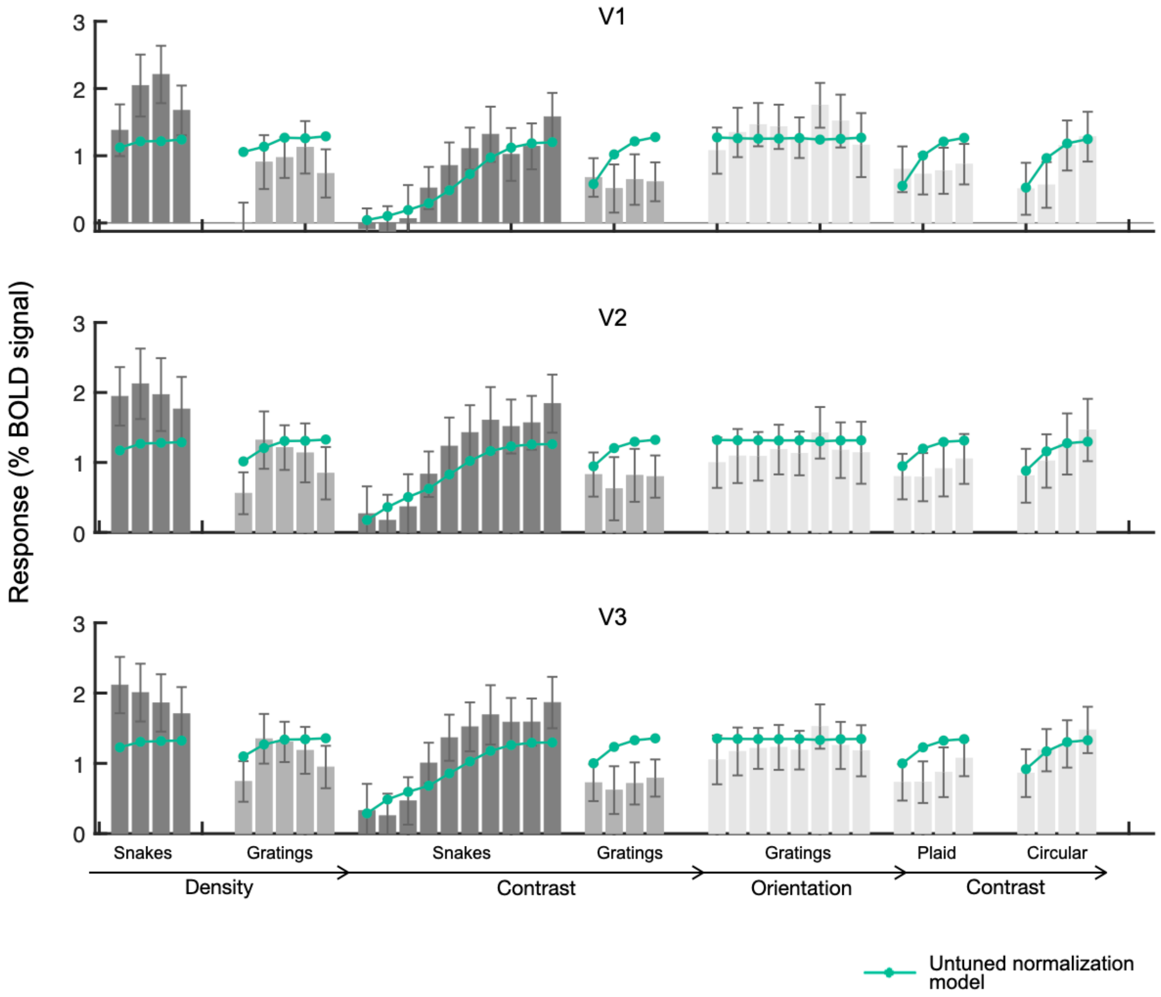

Fig D3: Responses and orientation-tuned normalization model fits for all stimuli, data set 3

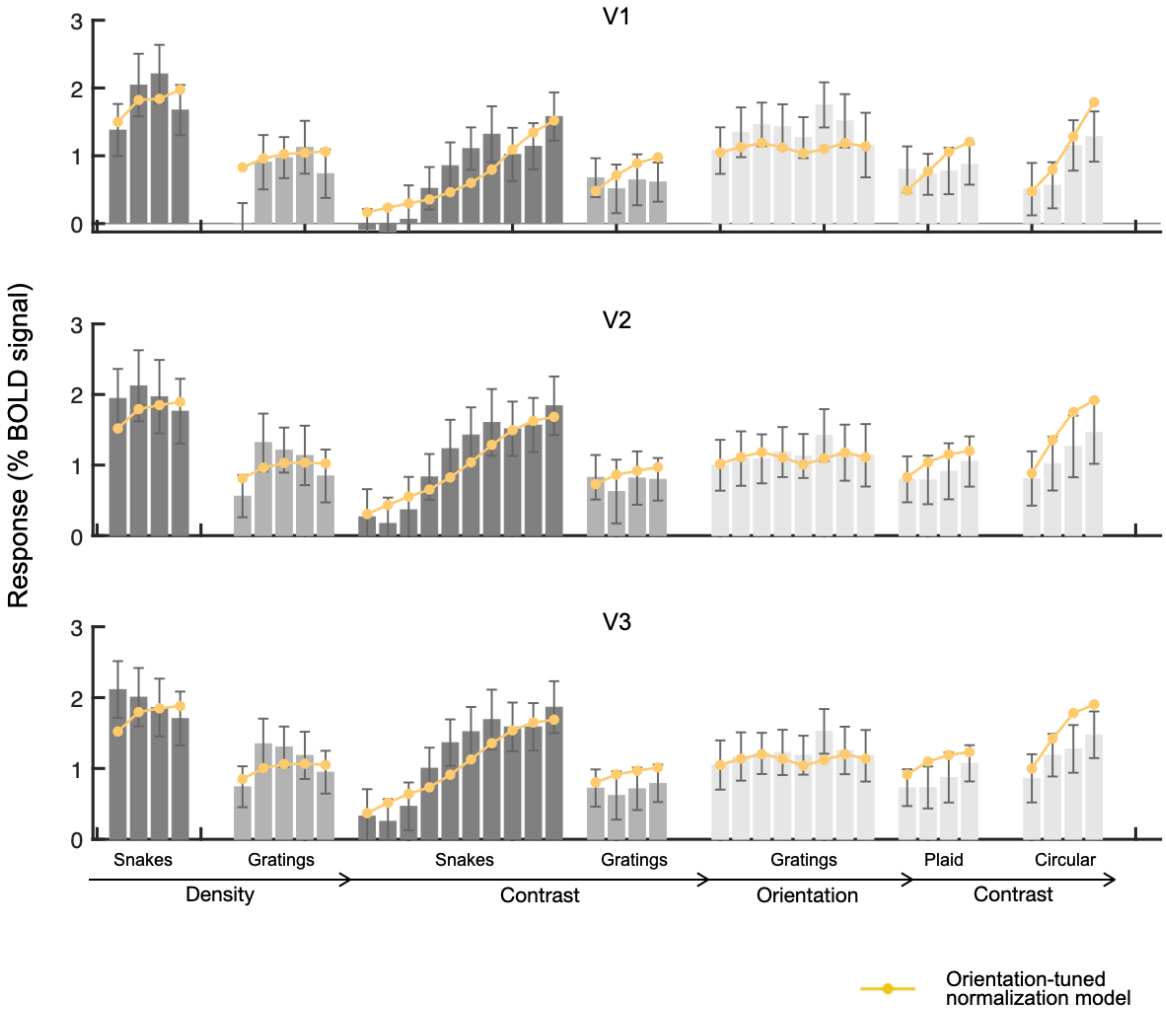

Fig D4: Responses and normalization by orientation anisotropy model fits for all stimuli, data set 3

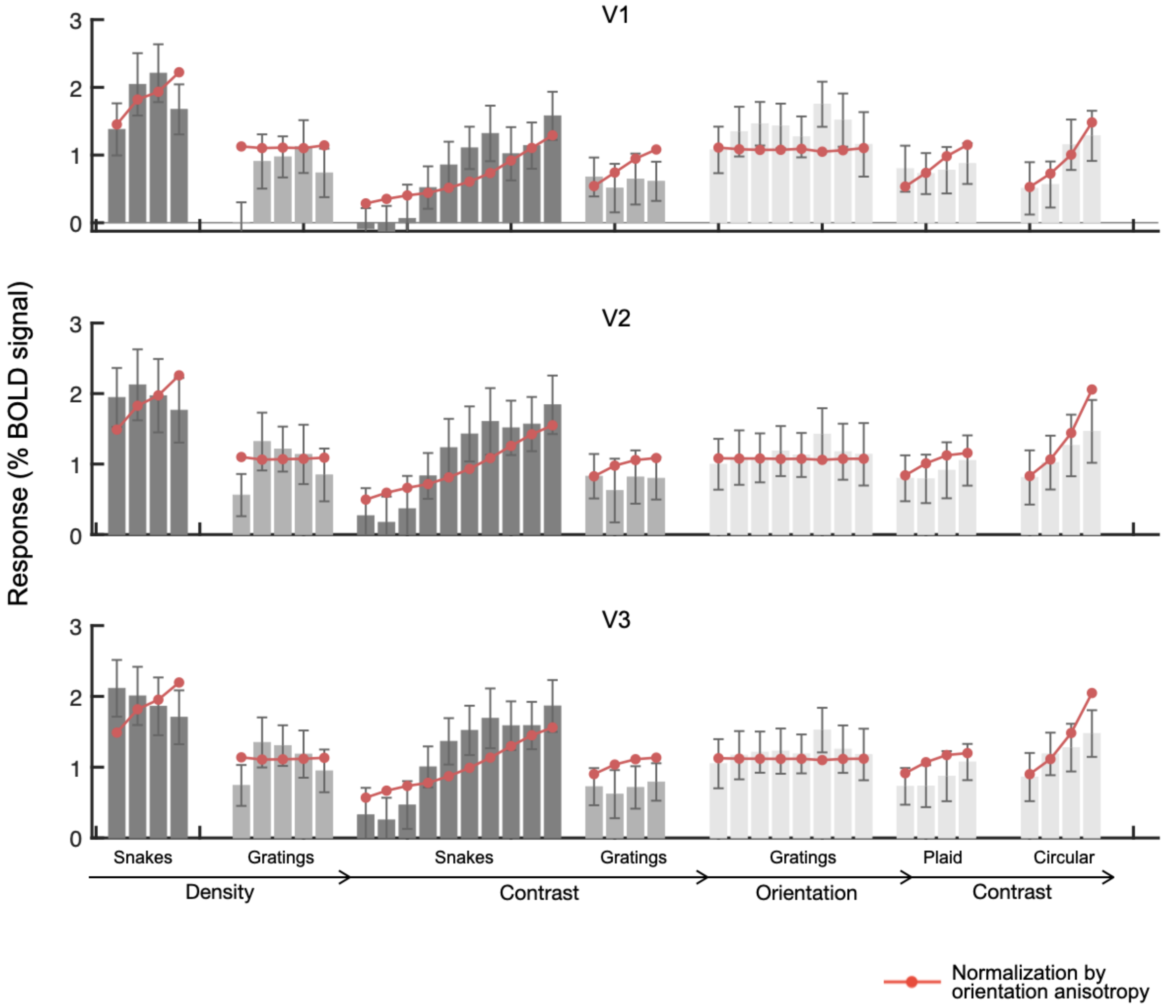

Fig E1: Responses and contrast energy model fits for all stimuli, data set 4

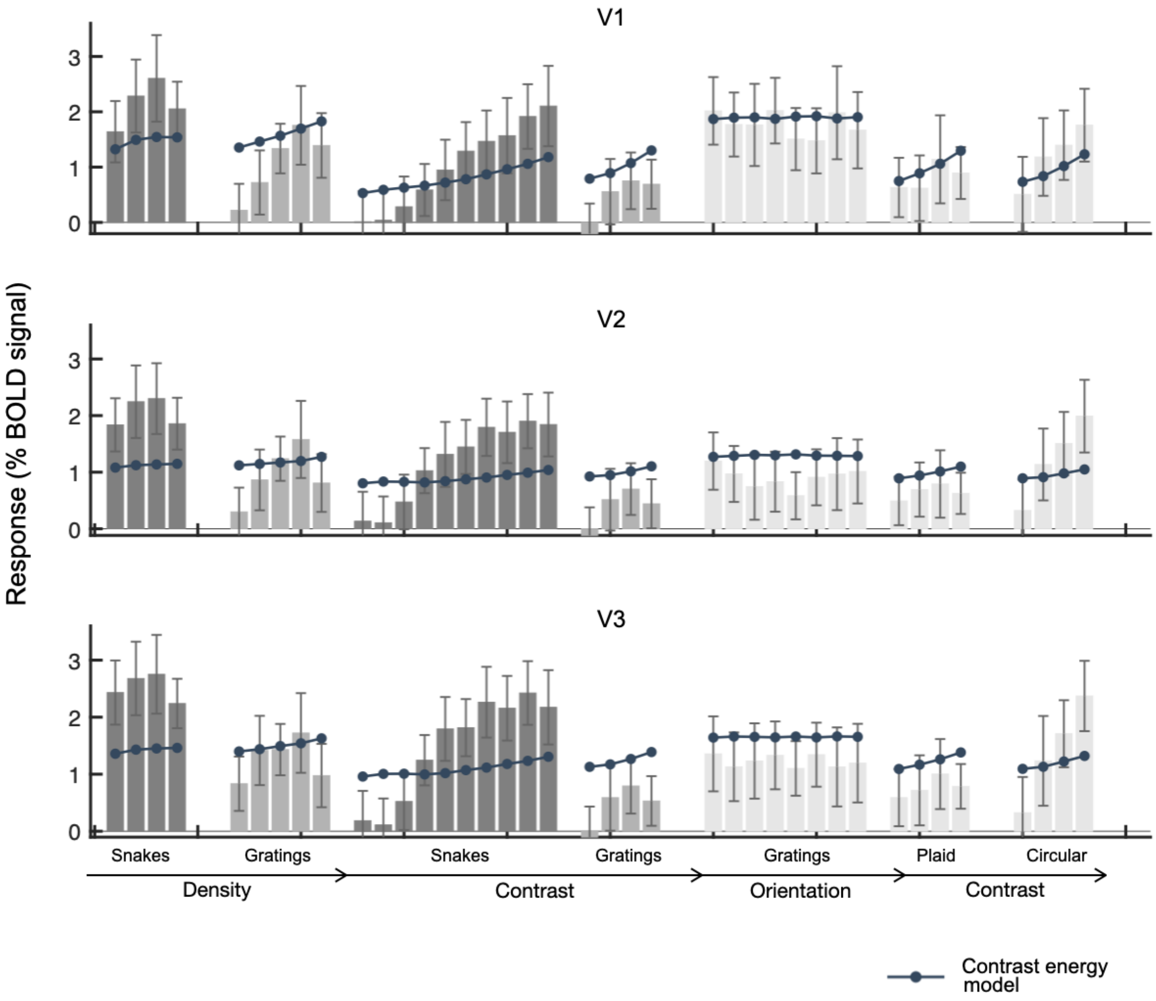

Fig E2: Responses and untuned normalization model fits for all stimuli, data set 4

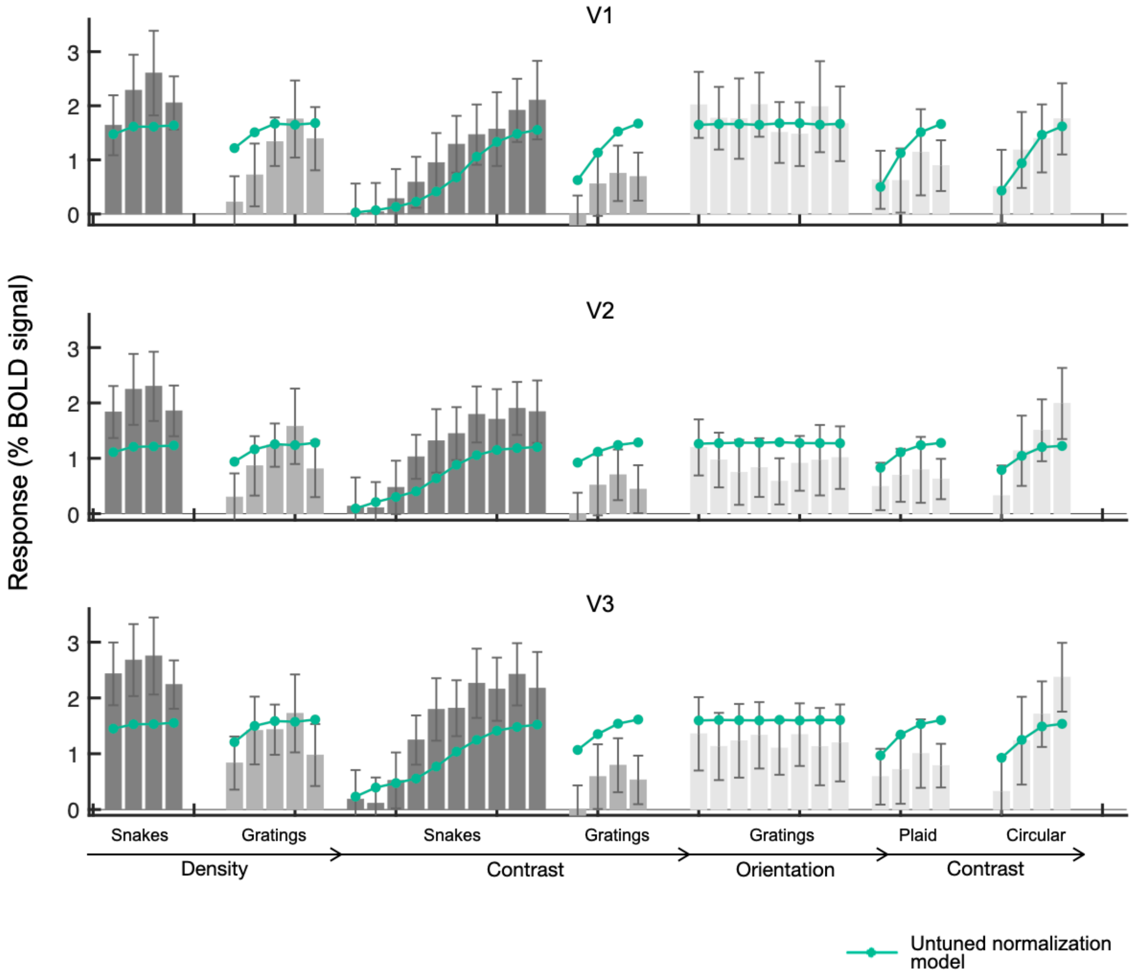

Fig E3: Responses and orientation-tuned normalization model fits for all stimuli, data set 4

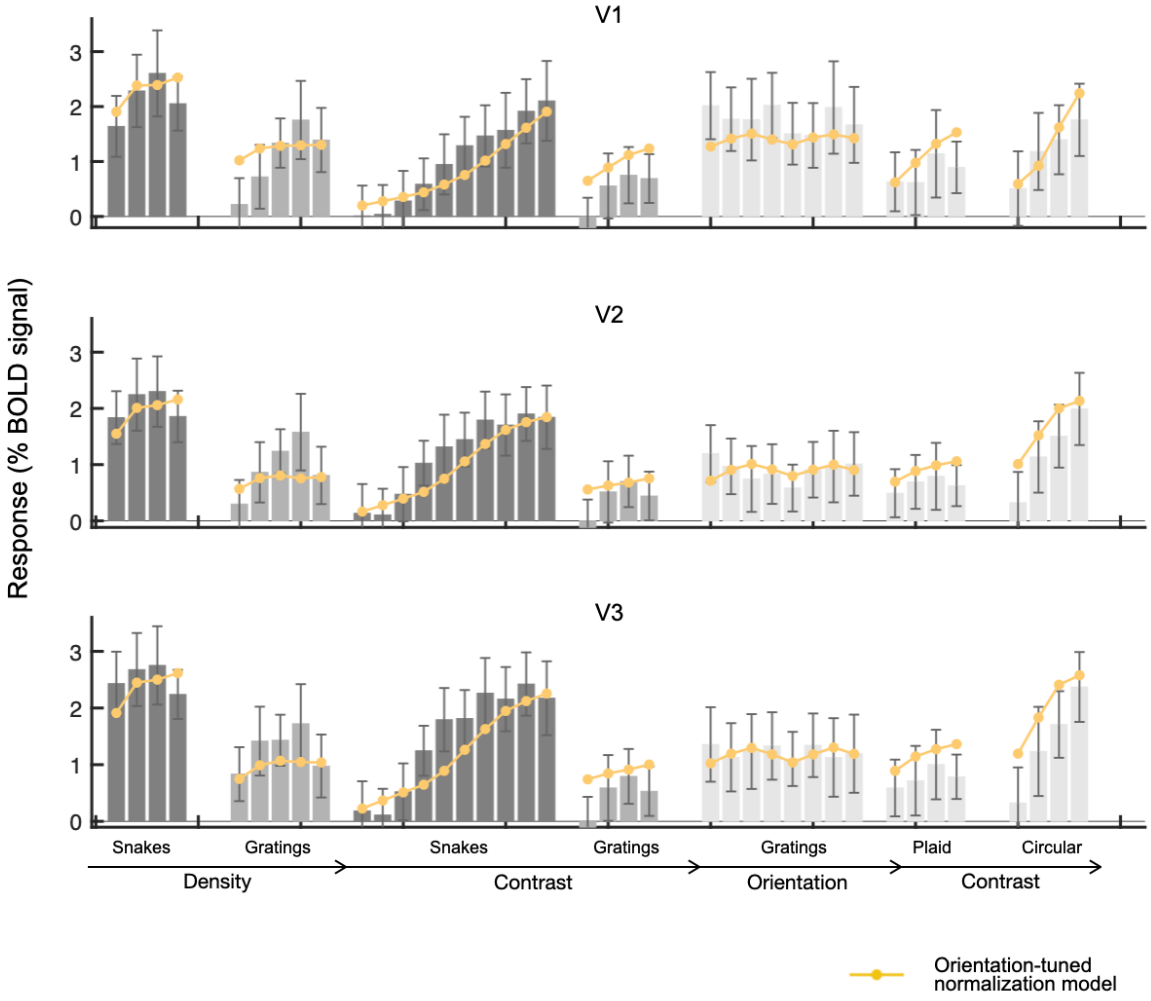

Fig E4: Responses and normalization by orientation anisotropy model fits for all stimuli, data set 4

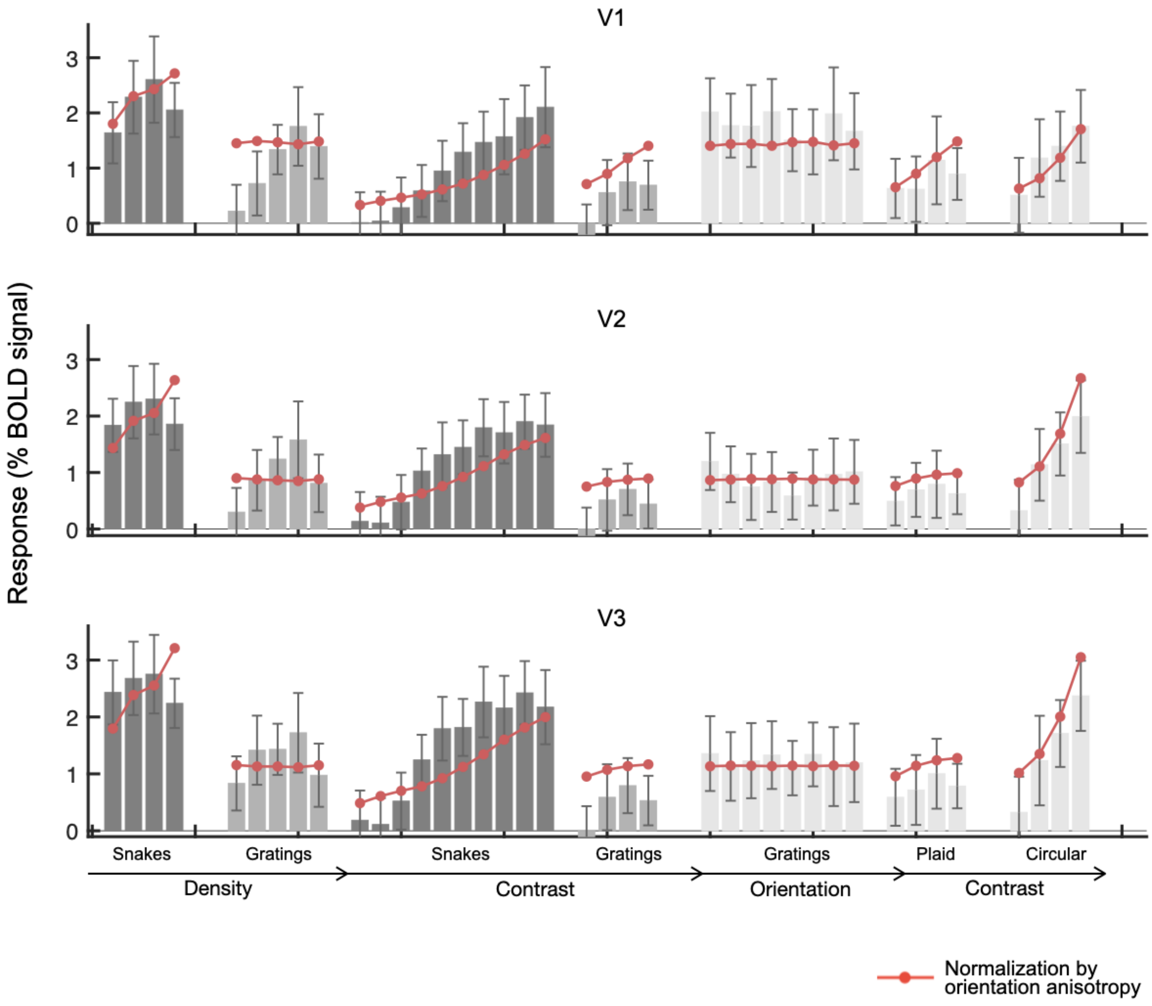

Supplement: S5 Appendix — (PDF) [file pcbi.1011704.s005.pdf]
